# Resonances at Fundamental and Harmonic Frequencies for Selective Imaging of Sine‐Wave Illuminated Reversibly Photoactivatable Labels

**DOI:** 10.1002/cphc.202200295

**Published:** 2022-09-21

**Authors:** Agnès Pellissier‐Tanon, Raja Chouket, Ruikang Zhang, Aliénor Lahlou, Agathe Espagne, Annie Lemarchand, Vincent Croquette, Ludovic Jullien, Thomas Le Saux

**Affiliations:** ^1^ PASTEUR Département de chimie École normale supérieure PSL University Sorbonne Université, CNRS 24, rue Lhomond 75005 Paris France; ^2^ Sony Computer Science Laboratories Paris France; ^3^ Sorbonne Université, Centre National de la Recherche Scientifique (CNRS) Laboratoire de Physique Théorique de la Matière Condensée (LPTMC) 4, Place Jussieu, Case Courrier 121 75252 Paris Cedex 05 France; ^4^ Laboratoire de Physique Statistique Département de Physique and Département de Biologie École normale supérieure PSL Research University, F- 75005 Paris France

**Keywords:** fluorescence imaging, harmonics, periodic forcing, resonance, reversibly photoactivatable label

## Abstract

We introduce HIGHLIGHT as a simple and general strategy to selectively image a reversibly photoactivatable fluorescent label associated with a given kinetics. The label is submitted to sine‐wave illumination of large amplitude, which generates oscillations of its concentration and fluorescence at higher harmonic frequencies. For singularizing a label, HIGHLIGHT uses specific frequencies and mean light intensities associated with resonances of the amplitudes of concentration and fluorescence oscillations at harmonic frequencies. Several non‐redundant resonant observables are simultaneously retrieved from a single experiment with phase‐sensitive detection. HIGHLIGHT is used for selective imaging of four spectrally similar fluorescent proteins that had not been discriminated so far. Moreover, labels out of targeted locations can be discarded in an inhomogeneous spatial profile of illumination. HIGHLIGHT opens roads for simplified optical setups at reduced cost and easier maintenance.

## Graphical abstract

**Improving label discrimination**: HIGHLIGHT (pHase‐sensItive imaGing of reversibly pHotoactivatable Labels after modulatIon of activatinG ligHT) is introduced as a simple and general strategy to selectively image a reversibly photoactivatable fluorescent label associated with given kinetics. HIGHLIGHT uses specific frequencies and mean light intensities associated with resonances of the amplitudes of concentration and fluorescence oscillations at harmonic frequencies. It is used for selective imaging of four spectrally similar fluorescent proteins that had not been discriminated so far.

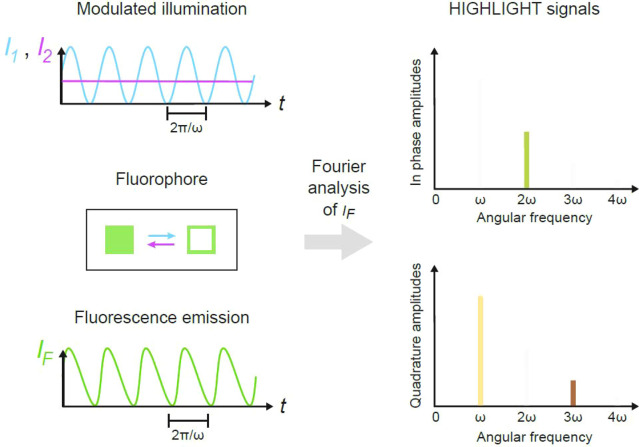

## Introduction

Analysis and imaging aim at identifying, quantifying, and localizing components in mixtures. Non‐invasive strategies are adopted in systems which should remain in their native state (e. g. living cells).^[1]^ A specific spectroscopic signature can be exploited to distinguish a target. However, complex media contain a large number of components often bearing similar atoms or functional groups, which hinders spectral target discrimination against the background. The thermodynamics (as used in titration) or the kinetics of a reaction between the target and a specific reagent can also be harnessed for further discrimination of spectrally similar components. Kinetics is especially attractive since it depends on more discriminative parameters, the rate constants, than thermodynamics, entirely determined by the equilibrium constants.[^1^, ^2^]

A kinetics‐based strategy has been implemented in light‐driven dynamic contrast, which leverages a reversibly photoactivatable label fused with the target. Light is used for both triggering the kinetics of the label photocycle and reading out.^[3]^ Dynamic contrast has found significant developments in multiplexed fluorescence imaging by exploiting the relaxation of reversibly excited[^4^, ^5^] or photoswitched labels.[^6^, ^7^, ^8^, ^9^, ^10^, ^11^, ^12^, ^13^, ^14^, ^15^] These imaging protocols provide appreciable selectivity but they still demand too wide a span of relaxation times for discrimination, which is inconsistent with imaging the large number of labelled biomolecules needed for advanced bioimaging.[^16^, ^17^]

An elegant way to reveal dynamics is to study the response of the system to a periodic forcing.[^18^, ^19^, ^20^, ^21^] Several imaging protocols[^4^, ^5^, ^9^, ^10^, ^18^] exploit phase‐sensitive analysis of the response at the fundamental angular frequency of a reversibly photoactivatable label submitted to periodic illumination.[^9^, ^10^] Selectivity for label discrimination can be improved by using the label response not only at the fundamental frequency but also at the harmonics. This approach has proven extremely fruitful in several fields such as chemical sensing,^[22]^ detection of atoms,^[23]^ subdiffraction‐limited imaging,[^24^, ^25^] electrochemical impedance spectroscopy,^[26]^ or fingerprinting reactive processes.[^27^, ^28^, ^29^, ^30^, ^31^]

In this paper, we derive the periodic fluorescence evolution of a reversibly photoactivatable fluorophore submitted to sine‐wave illumination of large amplitude. We demonstrate that a Fourier amplitude of concentration and fluorescence oscillations at each harmonic frequency exhibits at least one resonance of significant amplitude and determine the relationship observed at resonance between the kinetic properties of the label and experimentally‐controlled parameters. HIGHLIGHT (pHase‐sensItive imaGing of reversibly pHotoactivatable Labels after modulatIon of activatinG ligHT) harnesses this feature and yields several non‐redundant resonant observables from a single experiment with phase‐sensitive detection where the previous protocols (e. g. OPIOM[^9^, ^10^]) only yield one observable. Whereas the latter previous protocols are only concerned with the fluorescence response at the fundamental frequency of the periodic modulation of illumination, HIGHLIGHT deals with the fluorescence response at the harmonic frequencies, which enhances selectivity over previous protocols only relying on the fundamental angular frequency. Hence HIGHLIGHT is shown to achieve the discrimination of three reversibly photoswitchable fluorophores (Dronpa, Dronpa‐2, and rsFastLime), which remained out‐of‐reach with OPIOM. We eventually demonstrate that HIGHLIGHT is endowed with enhanced spatial contrast in non‐homogeneous light profiles.

## Results and Discussion

### HIGHLIGHT Principle

**Figure 1:**
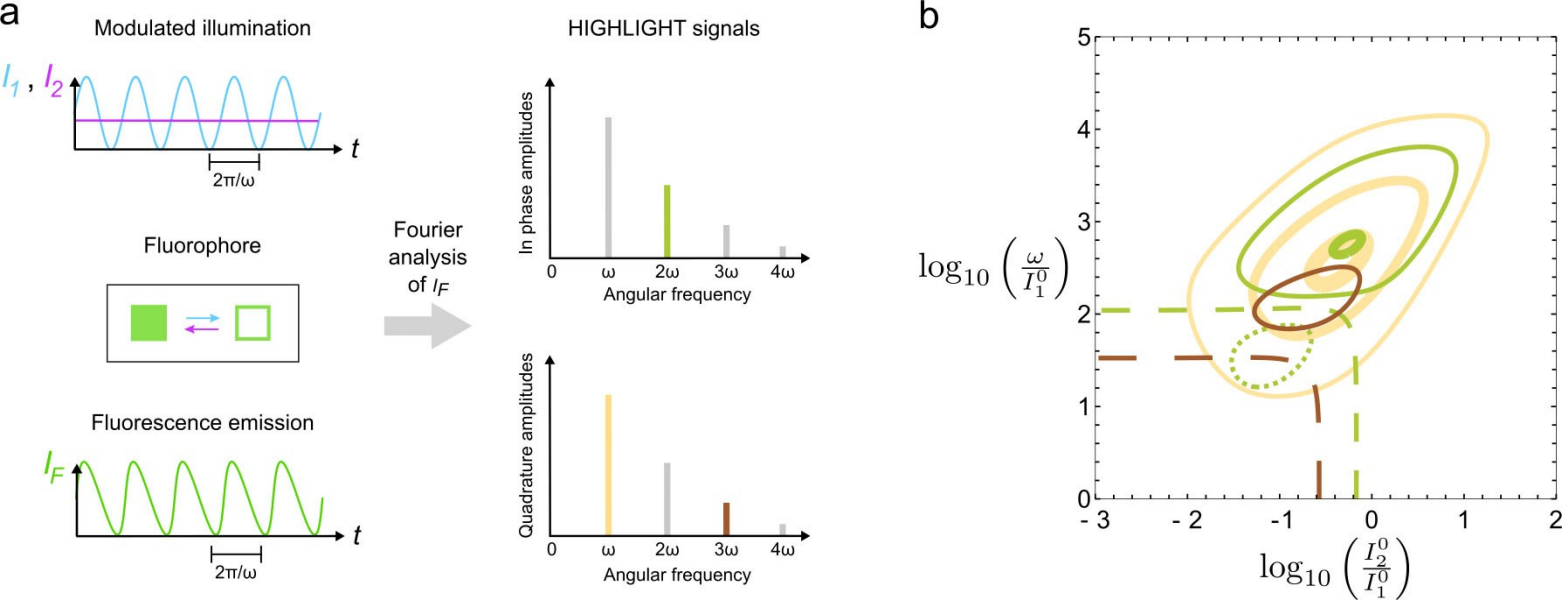
The HIGHLIGHT principle. a: Application of a sine‐wave illumination (here sine‐wave light at frequency ω and wavelength λ_1_ – in blue – and constant light at wavelength λ_2_ – in purple) on a reversibly photoactivatable fluorophore generates a periodic fluorescence signal, which provides quadrature and in‐phase amplitudes after Fourier analysis; b: Contour maps of the HIGHLIGHT‐n signals (n=1: yellow; n=2: green; n=3: brown) computed for Dronpa‐2 driven by sine‐wave light at λ_1_=480 nm and constant light at λ_2_=using the photochemical data shown in Tab. S5. Solid (dotted, resp.) lines are associated with positive (negative resp.) values. Dashed lines are zero lines. The HIGHLIGHT‐n signals are scaled by the HIGHLIGHT‐1 resonance: Thick lines correspond to amplitudes equal to 0.9, medium lines to 0.4, and thin lines to 0.1.

We consider a reversibly photoactivatable fluorophore engaged in a light‐driven exchange between two states differing by their brightness (see Figure 1a). The forward photoconversion results from illumination *I*_1_ at wavelength *λ*_1_ and the backward conversion is either spontaneous, e. g. by an emissive or thermally‐driven process, or photochemically governed under illumination *I*_2_ at wavelength *λ*_2_, as observed for the reversibly photoswitchable fluorescent proteins (RSFPs)[^32^, ^33^] used in the following.[^34^, ^35^, ^36^, ^37^] We analytically and numerically compute the evolution of the concentrations of the fluorophore states and the fluorescence signal under different illumination protocols involving sine‐wave light of angular frequency *ω* (see section S1.1 in Supporting Information): (i) light modulation at *λ*_1_ without light at *λ*_2_; (ii) light modulation at *λ*_1_ (*λ*_2_, resp.) with constant light at *λ*_2_ (*λ*_1_, resp.) (displayed in Figure 1a); (iii) antiphase modulation of both lights at *λ*_1_ and *λ*_2_. The photochemical rate constants being proportional to light intensity oscillate at frequency *ω*. Despite the linear dependence of the rate equations on the concentrations, the product between a photochemical rate constant and a concentration generates concentration oscillations not only at the fundamental frequency *ω* but also at harmonic frequencies. The harmonics in the fluorescence intensity result from the product of the light intensity and the concentration of a fluorophore. Fourier analysis is performed to extract the $n^{\mathrm{th}}$ ‐order amplitudes at each harmonic frequency nω for concentration and fluorescence intensity (see Figure 1a). The Fourier amplitudes are proportional to the total concentration of the photoactivatable fluorophore. Upon defining the phase of the Fourier amplitudes with respect to the exciting modulated light, the quadrature (for the odd orders) and in‐phase (for the even orders) amplitudes of both concentration and fluorescence oscillations exhibit at least one resonance in the space of the control parameters $\left(I_{1}^{0},\omega \right)$ in (i) or $\left(I_{2}^{0}/I_{1}^{0},\omega /I_{1}^{0}\right)$ in (ii) and (iii), where $I_{1}^{0}$ and $I_{2}^{0}$ are the mean light intensities. The resonant $n^{\mathrm{th}}$ ‐order Fourier amplitudes of fluorescence oscillation are further referred to as HIGHLIGHT‐*n* signals. For all illuminations, the HIGHLIGHT‐1 signal exhibits a single resonance peak, which is twice as high for illumination (iii) as for the other illuminations. From the second order, the HIGHLIGHT‐*n* signals have a single extremum or two extrema of opposite sign, leading to a line of vanishing amplitude. Far from extrema, the HIGHLIGHT‐*n* signals vanish. Importantly, the width of a resonance peak decreases as the order *n* of the harmonic increases without detrimental loss of absolute amplitude evaluated at the extremum (see Figure S6). Typically the HIGHLIGHT‐*n* signal decays by a factor of two when *n* is increased by one. The resonance conditions relate the control parameters and the kinetic properties of a given reversibly photoactivatable fluorophore. Specifically the resonant angular frequency *ω* is close to the inverse of the photoswitching relaxation time (see section S1.1 in Supporting Information). Imposing resonant conditions for a target label eliminates the contributions to the HIGHLIGHT‐*n* signals of non‐resonant spectrally interfering signals (e. g. other labels, autofluorescence, or ambient light). Hence, the HIGHLIGHT‐*n* signals provide a selective and quantitative imaging protocol.

The protocol (ii) with light modulation at *λ*_1_ is chosen for its well‐suited properties for selective imaging (see section S1.3 in Supporting Information). As a representative example of reversibly photoactivatable fluorophore for HIGHLIGHT evaluation, we choose the RSFP Dronpa‐2.^[38]^ As a negative photoswitcher, Dronpa‐2 is switched to a dark state by a blue light at $\lambda_{1}=480$ nm and back from the photoswitched dark state to a bright state by purple light at $\lambda_{2}=405$ nm. Figure 1b displays the computed maps of the HIGHLIGHT‐*n* signals (n=1 –3) in the $\left(I_{2}^{0}/I_{1}^{0},\omega /I_{1}^{0}\right)$ space. Interestingly, the resonance conditions of the positive peaks of HIGHLIGHT‐2 and HIGHLIGHT‐3 are close to the resonance condition of HIGHLIGHT‐1. The width of the peaks along the $\omega /I_{1}^{0}$ ‐axis significantly drops from HIGHLIGHT‐1 to HIGHLIGHT‐3 without severe decrease of peak amplitude. Hence a single acquisition with a same set of control parameters provides several independent and quantitative observables of increasing selectivity for fluorescence imaging.

### HIGHLIGHT in Action

HIGHLIGHT implementation requires pure sine‐wave light sources ensuring that non‐linearities revealed by the harmonics result from the response of the reversibly photoactivatable fluorophore and not from harmonic distortion of illumination. Considering the acquisition frequency of the camera and the resonant conditions and ranges of wavelength absorption for RSFPs, we aimed at generating pure sine‐wave modulation from Light Emitting Diodes (LEDs) at 480 and 405 nm at 1–50 Hz frequencies. Despite a waveform generator delivering a sinusoidal voltage with less than 0.075 % harmonic distortion, we noticed that the modulated light exhibited a detrimental level of harmonic distortion (see Figure S13a,b and S14a,b), presumably originating from temperature‐dependent brightness of the passively cooled LEDs. Hence we introduced a feedback control on the generator bringing harmonic distortion below 1 %. Equipped with reliable pure sine‐wave illumination, we could illustrate the opportunities provided by the set of HIGHLIGHT‐*n* observables for selective fluorescence imaging.

**Figure 2:**
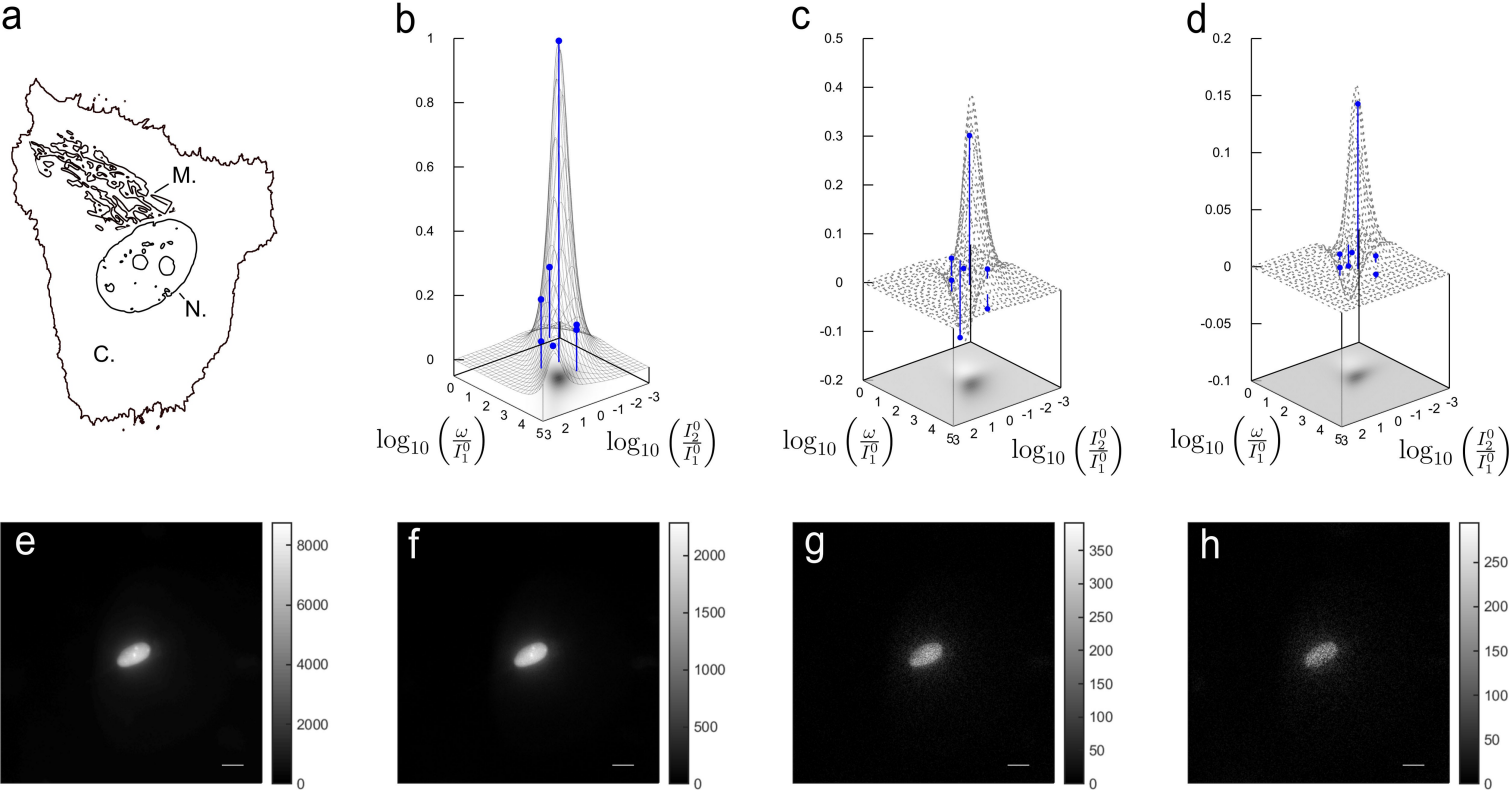
*HIGHLIGHT‐n discrimination maps using cell imaging*. **a**: Schematic representation of the imaged cell (N: nucleus; M: mitochondria); **b**–**d**: Experimental (blue disks) and theoretical (black dots) HIGHLIGHT‐1 (**b**), ‐2 (**c**), and ‐3 (**d**) signals scaled by HIGHLIGHT‐1 value at resonance in the ($I_{2}^{0}/I_{1}^{0}$ ,$\omega /I_{1}^{0}$ ) space for Dronpa‐2 under sine‐wave light at $\lambda_{1}=480$ nm and constant light at $\lambda_{2}=405$ nm; **e**–**h**: Pre‐HIGHLIGHT (**e**) and HIGHLIGHT‐1 (**f**), ‐2 (**g**), and ‐3 (**h**) images of Dronpa‐2. The level of the HIGHLIGHT‐*n* signals is displayed in the gray scale on the right of each figure. System: Fixed U2OS cells expressing H2B‐Dronpa‐2 (at the nucleus). The images were recorded at 298 K with scale bars: 10 μm. The control parameters are set at the resonance of HIGHLIGHT‐1 for Dronpa‐2. See Tab. S6 in Supporting Information for the acquisition conditions.

We first experimentally evaluated the theoretical predictions of the discriminative map displayed in Figure 1b. We imaged fixed nucleus‐labeled U2OS cells (see Figure 2a) expressing H2B‐Dronpa‐2 using a home‐built epifluorescence microscope delivering spatially homogeneous sine‐wave light at $\lambda_{1}=480$ nm and constant light at $\lambda_{2}=405$ nm under fulfilling the HIGHLIGHT‐1 resonance conditions using reported photochemical information (see Figure 2b–h).[^10^, ^39^] Fourier transform was used to extract the in‐phase and quadrature components leading to HIGHLIGHT‐*n* images with satisfactory signal‐to‐noise ratios $S/N\left(n\right)$ up to n=3 : $S/N\left(1\right)=10.2$ , $S/N\left(2\right)=11.4$ , and $S/N\left(3\right)=6.1$ (see also Figure S16a–d and Figure S17a–d for Dronpa‐3^[40]^‐ and rsFastLime[^38^, ^41^]‐labeled cells, respectively). The HIGHLIGHT‐1–3 signals agreed with the theoretical predictions. The measured HIGHLIGHT‐2 and ‐3 signals scaled by the HIGHLIGHT‐1 signal were 0.31±0.12 and 0.14±0.16 when 0.35 and 0.10 could be expected, respectively. The signals HIGHLIGHT‐1–3 obtained at conditions deviating from the HIGHLIGHT‐1 resonance by an order of magnitude along each axis were in agreement with the predictions. In particular, the expected decrease of the width of the peak along the the $\omega /I_{1}^{0}$ ‐axis from HIGHLIGHT‐1 to HIGHLIGHT‐3 was confirmed. Eventually, we observed the vanishing of the HIGHLIGHT‐2 and HIGHLIGHT‐3 signals and the change of their sign for smaller control parameters than the resonant values along the diagonal.

**Figure 3:**
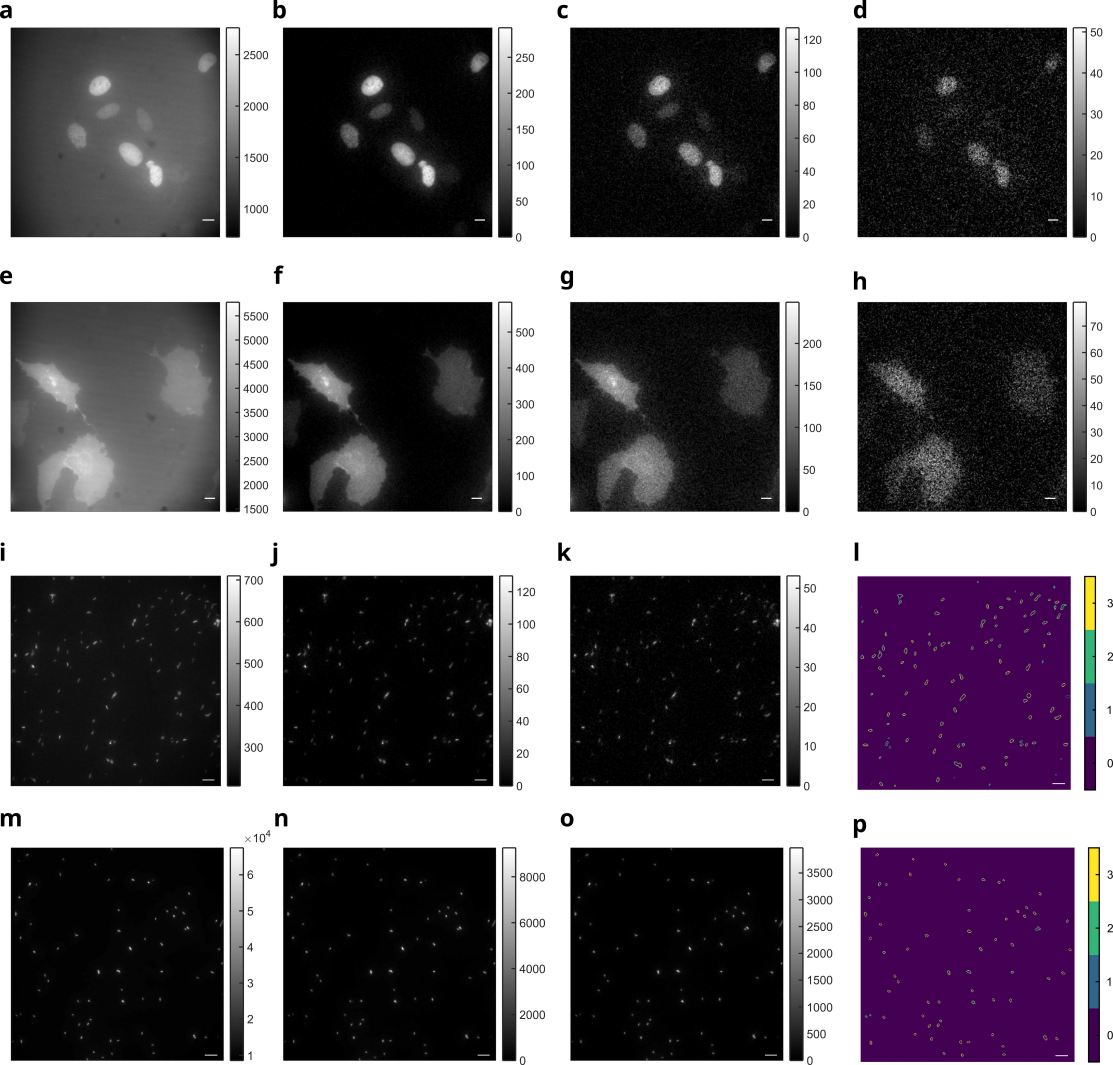
*HIGHLIGHT‐n imaging in living cells and in bacteria*. **a**–**h**: Pre‐HIGHLIGHT (**a,e**) and HIGHLIGHT‐1 (**b,f**), ‐2 (**c,g**), and ‐3 (**d,h**) images of Dronpa‐2‐ (**a**–**d**) and rsFastLime‐ (**e**–**h**) labeled living cells. The level of the HIGHLIGHT‐*n* signals is displayed in the gray scale on the right of each figure. System: Living U2OS cells expressing H2B‐Dronpa‐2 (at the nucleus) or Lyn11‐rsFastLime (at the membrane); **i**–**p**: Pre‐HIGHLIGHT (**i**,**m**) and HIGHLIGHT‐1 (**j**,**n**), ‐2 (**k**,**o**) images of cells expressing either Dronpa‐2 (**i**–**k**) or Dronpa (**m**–**o**). The level of the HIGHLIGHT‐*n* signals is displayed in the gray scale on the right of each figure. After segmentation of bacteria from the Pre‐HIGHLIGHT, HIGHLIGHT‐1and HIGHLIGHT‐2 images (on **i**–**k** and **m**–**o** images), the contour of segmented bacteria were extracted and the number of colocalizations of the contours is displayed in **l** and **p** images using the Pre‐HIGHLIGHT image (**i** or **m**) as reference. It ranged from 0 indicating no contour at the pixel to 3 indicating colocalization for all the extracted contours; 1 indicating a detected contour in the Pre‐HIGHLIGHT image only. System: Living *E. Coli* cells expressing Dronpa‐2 (**i**–**l**) or Dronpa (**m**–**p**). A median filter 3×3 was applied to obtain the final HIGHLIGHT‐1 and HIGHLIGHT‐2 images. The images were recorded at 298 K with scale bars: 10 μm. The control parameters are set at the resonance of HIGHLIGHT‐1 for Dronpa‐2 (**a**–**d**,**i**–**k**), rsFastLime (**e**–**h**), and Dronpa (**m**–**p**). See Tab. S6 in Supporting Information for the acquisition conditions.

We then showed that the HIGHLIGHT discriminative maps validated on fixed cells were as well relevant to image live systems with our home‐built epifluorescence microscope. We first imaged living U2OS cells expressing either H2B‐Dronpa‐2 or Lyn11‐rsFastLime with the epifluorescence microscope under sine‐wave light at $\lambda_{1}=480$ nm and constant light at $\lambda_{2}=405$ nm at their HIGHLIGHT‐1 resonance conditions using reported photochemical information.[^10^, ^39^] As displayed in Figure 3a–h, we could get HIGHLIGHT‐1–3 images very much similar to the ones obtained with the fixed U2OS cells under the same conditions of acquisition (see Figure 2e–h). In particular, we did not encounter any significant detrimental issue of image blur (that may arise from cell motion) nor of phototoxicity (from dual illumination at 405 and 480 nm) during the 10 s‐long HIGHLIGHT acquisition which we used for imaging Dronpa‐2 and rs‐FastLime. Yet, such issues should have to be considered when using slower photoswitching RSFPs (e. g. Dronpa), which necessitate longer HIGHLIGHT acquisitions. Then we addressed the possible dispersion and the consistency of the HIGHLIGHT response by imaging numerous living *E. Coli* bacteria expressing Dronpa‐2 and Dronpa under sine‐wave light at $\lambda_{1}=480$ nm and constant light at $\lambda_{2}=405$ nm at their HIGHLIGHT‐1 resonance conditions using reported photochemical information (see Figure 3i–p).[^10^, ^39^] Fourier transform was used to extract the HIGHLIGHT‐n images and the cells were further segmented using a previously reported algorithm.^[39]^ For the dimmer Dronpa‐2 (see Figure 3i–l), the regions segmented from the HIGHLIGHT‐1 and HIGHLIGHT‐2 images satisfactorily shared 98 % and 87 % of their pixels with the Pre‐HIGHLIGHT image respectively. For Dronpa (see Figure 3m–p), the regions segmented from the HIGHLIGHT‐1 and HIGHLIGHT‐2 images shared 98 % of their pixels with the Pre‐HIGHLIGHT image. Hence we did not notice any significantly detrimental dispersion of the HIGHLIGHT response among the population of Dronpa‐2‐ or Dronpa‐labeled bacteria, which is in line with our previous results.^[39]^

After evidencing the relevance of the HIGHLIGHT discrimination maps in fixed and live cells, we showed that HIGHLIGHT provides access to quantitative imaging of a targeted RSFP against a background of spectrally interfering fluorophores. To address this property, we filled six chambers of a microfluidic device with solutions containing mixtures of Dronpa‐2 and the spectrally interfering non‐photoactive fluorescent protein EGFP in different proportions (see Figure S18). Two separate sets of images were collected under sine‐wave light at $\lambda_{1}=480$ nm and constant light at $\lambda_{2}=405$ nm at HIGHLIGHT‐1 resonance for Dronpa‐2: One set results from averaging the fluorescence evolution and similar to an image recorded under constant illumination (hereafter called pre‐HIGHLIGHT image) and the second set corresponds to the processed HIGHLIGHT image. Dronpa‐2 fluorescence emission could be detected in both pre‐HIGHLIGHT and HIGHLIGHT images. In contrast, as expected from the absence of any HIGHLIGHT contribution in its fluorescence emission, EGFP gave no signal on the HIGHLIGHT‐1 and HIGHLIGHT‐2 image, thus demonstrating the expected selective imaging of Dronpa‐2 with HIGHLIGHT. Moreover, the five chambers with Dronpa‐2 showed relative intensities directly reflecting their concentration, which confirmed the theoretical prediction that the HIGHLIGHT signal is proportional to the probe concentration. A similar result was obtained in microscopy of mammalian cells expressing both Dronpa‐2 and EGFP. Whereas the contributions of Dronpa‐2 and EGFP were superimposed in the pre‐HIGHLIGHT image (Figure S19a), only Dronpa‐2 contributed to the HIGHLIGHT‐1 and HIGHLIGHT‐2 images as anticipated from the absence of any resonant photoswitching behavior for EGFP (Figure S19b,c). Hence, HIGHLIGHT gives access to quantitative fluorescence imaging upon efficiently suppressing the signal from spectrally interfering non‐photoactive fluorophores.

**Figure 4:**
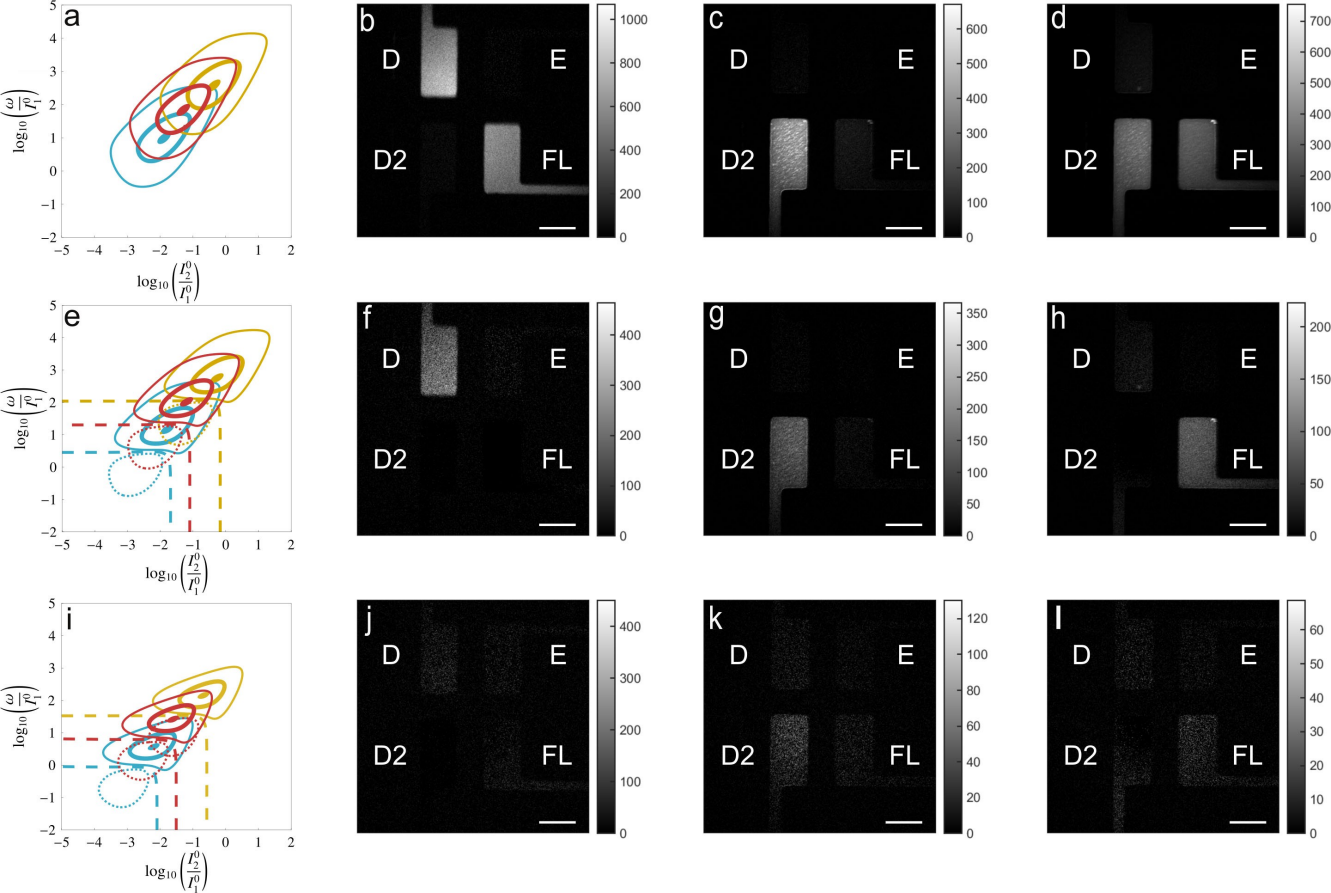
*HIGHLIGHT discriminates four distinct spectrally similar fluorescent proteins*. **a**,**e**,**i**: Contour maps of the scaled HIGHLIGHT‐1 (**a**), ‐2 (**e**), and ‐3 (**i**) signals under sine‐wave light at $\lambda_{1}=480$ nm and constant light at $\lambda_{2}=405$ nm for Dronpa (light blue), rsFastLime (red) and Dronpa‐2 (brown) in the ($I_{2}^{0}/I_{1}^{0}$ ,$\omega /I_{1}^{0}$ ) space using the data shown in Tab. S5 (see Supporting Information). Solid (dotted, resp.) lines are associated with positive (negative, resp.) values. Dashed lines are zero lines. The HIGHLIGHT‐*n* signals are scaled by the HIGHLIGHT‐1 value at resonance: Thick lines correspond to amplitudes equal to 0.9, medium lines to 0.4, and thin lines to 0.1; HIGHLIGHT‐*n* (n=1 : **b**–**d**; n=2 : **f**–**h**; n=3 : **i**–**l**) images of a four‐chamber microsystem filled with solutions of Dronpa (D; 2 μM), Dronpa‐2 (D2; 8 μM), rsFastLime (FL; 5 μM), and EGFP (E; 5 μM) in pH 7.4 BSA‐PBS buffer. The images were recorded at 298 K. Scale bars: 100 μm. The control parameters are set at the resonance of HIGHLIGHT‐1 Dronpa (**b**,**f**,**j**), for Dronpa‐2 (**c**,**g**,**k**), and rsFastLime (**d**,**h**,**l**). See Tab. S6 in Supporting Information for the acquisition conditions.

Then we showed that spectrally similar RFSPs (see Figure S20) possessing different kinetic properties could be selectively imaged with HIGHLIGHT. We specifically examined the negative photoswitchers Dronpa,^[42]^ Dronpa‐2,^[38]^ and rsFastLime,[^38^, ^41^] which could not be independently imaged using Speed OPIOM.^[10]^ The theoretical predictions for the HIGHLIGHT‐*n* maps of Dronpa and rsFastLime were first determined using light modulation at $\lambda_{1}=480$ nm and constant light at $\lambda_{2}=405$ nm (see Figure 4a,e,i). Then we imaged a four‐chamber microfluidic device, each chamber being filled with a solution of one fluorophore, Dronpa, Dronpa‐2, rsFastLime, or EGFP. Three fluorescence evolutions were recorded upon fulfilling the HIGHLIGHT‐1 resonance conditions of the three RSFPs, Dronpa, Dronpa‐2, and rsFastLime. As expected, the EGFP chamber was dark in all HIGHLIGHT‐*n* images. Whereas the HIGHLIGHT‐1 image recorded at resonance for Dronpa‐2 could almost eliminate the interfering contributions of the two other RSFPs (see Figure 4c), the HIGHLIGHT‐1 images recorded at resonance for Dronpa and rsFastLime encountered strong interferences from rsFastLime and Dronpa‐2 respectively (see Figure 4b and d) as anticipated from significant overlaps in Figure 4a. This result was expected from the similarity between the HIGHLIGHT‐1 and Speed OPIOM imaging protocols. In contrast, HIGHLIGHT‐2 could selectively image the three RSFPs under their respective conditions of HIGHLIGHT‐1 resonance (see Figure 4f–h). This result was predicted by the theoretical maps showing that the point associated with the HIGHLIGHT‐1 resonant parameter values of Dronpa and rsFastLime lies on the HIGHLIGHT‐2 zero line of rsFastLime and Dronpa‐2 respectively (see Figure 4a and e, and d and h).

The discrimination power of HIGHLIGHT‐2 was further evidenced in living and fixed cells. rsFastLime and Dronpa‐2 have been first discriminated with HIGHLIGHT‐2 in fixed and living U2OS cells (see Figure S21). Whereas the Pre‐HIGHLIGHT (Figure S21a,d,g,j) and HIGHLIGHT‐1 (Figure S21b,e,h,k) images evidenced overlap of the signals from both RSFPs, HIGHLIGHT‐2 unambiguously distinguished rsFastLime at the cell membrane from Dronpa‐2 at the nucleus (Figure S21c,f,i,l). Similar observations have been made for discriminating Dronpa and Dronpa‐2 in living bacteria (Figure S22). Hence, when adopting the HIGHLIGHT‐1 resonance conditions for acquisition, both HIGHLIGHT‐1 and HIGHLIGHT‐2 signals can be easily accessed. Moreover HIGHLIGHT‐2 emerged as the most attractive observable balancing selectivity with sensitivity for fluorescence imaging of reversibly photoactivatable fluorophores.

**Figure 5:**
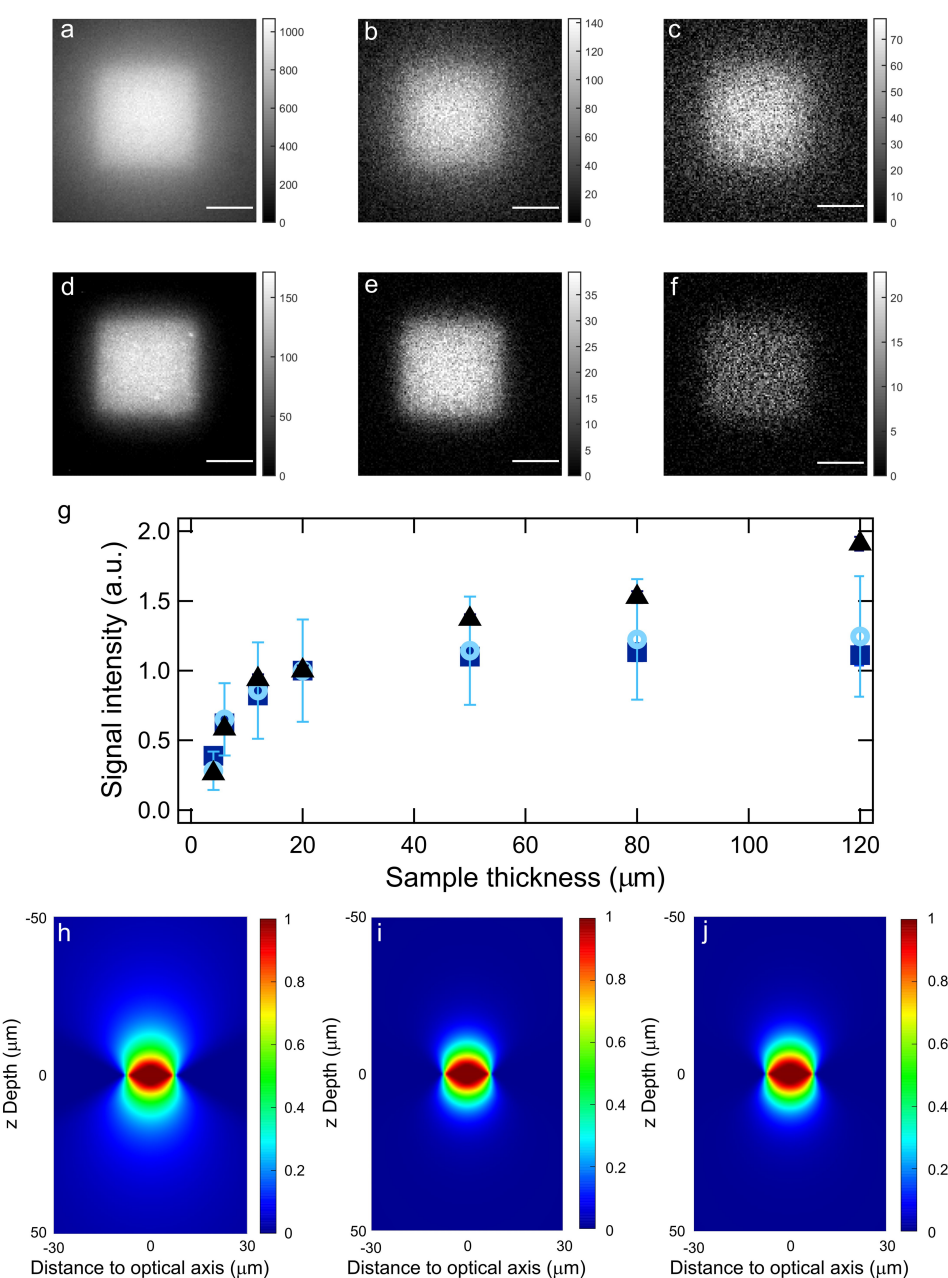
*HIGHLIGHT capability to enhance spatial contrast in non‐homogeneous light profiles*. Pre‐HIGHLIGHT (**a**,**d**), HIGHLIGHT‐1 (**b**,**e**), ‐2 (**c**,**f**) images of a 10‐μM Dronpa‐2 solution of thickness 120 μm (**a**–**c**) and 4 μm (**d**–**f**) obtained with an optical setup using coaxial light profiles spatially decaying over 10 μm from a homogeneously illuminated 15‐μm edge octahedron. Scale bar: 10 μm; **g**: Pre‐HIGHLIGHT (black triangles), HIGHLIGHT‐1 (deep blue squares), ‐2 (light blue circles) signals versus thickness *z* of the sample for a 10‐μM Dronpa‐2 solution. The mean signal intensities and the error bars are obtained using spatial averages of the images over a 6‐μm square; Spatial dependence of the scaled Pre‐HIGHLIGHT (**h**), HIGHLIGHT‐1 (**i**), and ‐2 (**j**) signals after introducing the light profiles in the analytical expressions of the HIGHLIGHT signals. See Tab. S6 in Supporting Information for the acquisition conditions with sine‐wave light at $\lambda_{1}=480$ nm and constant light at $\lambda_{2}=405$ nm.

In addition to selective imaging performances, HIGHLIGHT is endowed with enhanced spatial contrast in non‐homogeneous light profiles. Imposing an inhomogeneous spatial profile of illumination allows the HIGHLIGHT resonance conditions to be met only locally, which enables us to easily discard fluorophores out of targeted locations. To evidence this attractive feature which could not be evidenced with our previous epifluorescence microscope delivering homogeneous illumination, we built another epi‐fluorescence imaging system (see subsection S2.6 in the Supporting Information) that delivers coaxial light profiles at 480 and 405 nm spatially decaying over 10 μm from a homogeneously illuminated 15‐μm edge octahedron located at the focal plane. This setup was used to image layers of variable thickness of a Dronpa‐2 solution with sine‐wave light at $\lambda_{1}=480$ nm and constant light at $\lambda_{2}=405$ nm by choosing the values of the control parameters in the octahedron to meet the HIGHLIGHT‐1 resonance conditions. Figure 5a–f displays the Pre‐HIGHLIGHT, HIGHLIGHT‐1, and ‐2 images for 4‐μm and 120‐μm thick samples. For the 4‐μm thick sample, the three images do not exhibit any halo around the bright central square as anticipated from the absence of any fluorescence emission out of the focal plane. For the 120‐μm thick sample, the Pre‐HIGHLIGHT image yields a bright square surrounded by a strong halo, which originates from the contribution of the solution located above and below the octahedron. The HIGHLIGHT‐1 and HIGHLIGHT‐2 images provide a better defined square with a less pronounced halo since Dronpa‐2 experiences out‐of‐resonance conditions above and below the octahedron. To be quantitative, we measured the Pre‐HIGHLIGHT, HIGHLIGHT‐1, ‐2 signals as a function of the sample thickness *z*. From z=0 to 15 μm, both Pre‐HIGHLIGHT and HIGHLIGHT‐1, ‐2 signals linearly increase as expected from homogeneous light distribution within the octahedron (see Figure 5g). Beyond 15 μm, the Pre‐HIGHLIGHT signal still increases with increasing *z*. In contrast, the HIGHLIGHT‐1, ‐2 signals saturate, which demonstrates their capability to enhance spatial contrast in non‐homogeneous light profiles. These conclusions were confirmed when taking into account the spatial dependence of the light intensities in the analytical expressions of the HIGHLIGHT signals. Figs. 5 h–j show that the HIGHLIGHT‐1 and ‐2 signals are more focused than the Pre‐HIGHLIGHT signal. Similar results were obtained with modulated light at $\lambda_{2}=405$ nm and constant light at $\lambda_{1}=480$ nm (see Figure S23a–g).

## Concluding Remarks

The development of HIGHLIGHT has been motivated by improving dynamic contrast of reversibly photoactivatable fluorophores engaged in a two‐state exchange.[^10^, ^43^] We harness the resonant behavior of the HIGHLIGHT‐*n* signals defined as $n^{\mathrm{th}}$ order Fourier amplitudes obtained by phase‐sensitive Fourier analysis of fluorescence evolution under periodic illumination. We more specifically adopted pure sine‐wave illumination, which is favorable for discrimination with excellent signal‐to‐noise ratio from lock‐in amplification. Provided that simple photochemical experiments[^9^, ^10^] have revealed the intensity dependence of the rate constants, the parameters that control illumination can be tuned to maximize the HIGHLIGHT‐*n* signals of a targeted fluorophore and minimize the contributions of spectrally similar mixture components. The control parameter dependence of the HIGHLIGHT‐*n* signals predicted by the theoretical analysis has been experimentally confirmed.

In particular, the HIGHLIGHT‐1 signal has a single extremum whereas the HIGHLIGHT‐2 signal has two narrower and lower resonance peaks separated by a zero line in the space of control parameters. HIGHLIGHT‐1 and Speed‐OPIOM[^9^, ^10^] both harness the $1^{\mathrm{st}}$ ‐order quadrature Fourier amplitude of fluorescence oscillations. Whereas HIGHLIGHT‐1 and Speed‐OPIOM efficiently fought against autofluorescence, they both failed to distinguish spectrally similar reversibly photoactivatable fluorophores with closely located resonances. Under such demanding situations, we demonstrated the added value of HIGHLIGHT‐2. Without loosing much signal and signal‐to‐noise ratio with respect to HIGHLIGHT‐1, we independently imaged three RSFPs with similar resonances using the narrower resonance peak and the zero lines of HIGHLIGHT‐2.

Moreover we showed that HIGHLIGHT‐1 and ‐2 can enhance spatial contrast in non‐homogeneous light profiles,[^44^, ^45^] which is favorable to the implementation of 3D‐fluorescence imaging. Interestingly, independent light beam shaping at both wavelengths is not required (like in REversible Saturable OpticaL Fluorescence Transitions (RESOLFT)^[46]^ for instance) and only the generation of any non uniform light profiles is necessary. The simple illumination scheme of HIGHLIGHT may find promising applications in the context of light scanning (e. g. confocal microscopy) and could simplify the optical setup, reduce its cost, and facilitate its maintenance.

In HIGHLIGHT, the parameters of acquisition are fixed by resonance conditions, which involve the photoswitching cross sections associated to with back and forth photoswitching between state of distinct brightness as well as light intensities at the targeted location. These photoswitching cross sections may be anticipated to depend on several physicochemical parameters (e. g. pH). However, we have previously evidenced the robustness of the photoswitching cross sections of reversibly photoswitchable fluorescent proteins in aqueous solution, polymer matrices, and a battery of live and fixed samples (bacteria, eucaryotic cells from animals and plants, leaves and roots of live plants).[^9^, ^10^, ^39^, ^47^, ^48^, ^49^] The resonance conditions also suggest that HIGHLIGHT application will be at the most favorable in transparent media, where the light intensity can be reliably estimated. Eventually, one should notice that the minimal duration of a HIGHLIGHT image experiences an intrinsic limitation by the period of the modulated illumination.

HIGHLIGHT has been applied to fluorophores engaged in a two‐state exchange but it can also be directly applied to various imaging methods associated with observables that linearly depends on the concentration. In particular, it will benefit from the active development of reversibly photoswitchable fluorophores,[^32^, ^36^, ^50^] which exhibit light‐driven switch between states of different brightness upon illumination at one or two different wavelengths and which have been recently popularized by super‐resolution microscopies.[^46^, ^51^, ^52^, ^53^]

Eventually, the principle of HIGHLIGHT can be implemented in the case of more complex mechanisms.^[54]^ When the mechanism is known, resonance conditions can be determined leading to analytical expressions, which can be straightforwardly used to set the experimental conditions. For unknown mechanisms, empirical determination of resonances remains possible. Eventually, it is worth noting that complex mechanisms offer a large number of control parameters and the possibility to tune different rate constants and get a better selectivity for label discrimination.

## Supporting Information Appendix (SI)

HIGHLIGHT theory, Experimental Section, Figs. S1 to S21, Tables S1 to S6.

## Conflict of interest

A. P.‐T., R.C., R.Z., A.E., A. L., V. C., L. J. and T.L.S. have a patent relating to aspects of the work described in this manuscript.

## Supporting information

As a service to our authors and readers, this journal provides supporting information supplied by the authors. Such materials are peer reviewed and may be re‐organized for online delivery, but are not copy‐edited or typeset. Technical support issues arising from supporting information (other than missing files) should be addressed to the authors.

Supporting Information
