# Supplementary material for: Resonances at Fundamental and Harmonic Frequencies for Selective Imaging of Sine‐Wave Illuminated Reversibly Photoactivatable Labels
Source: Chemphyschem. 2022 Sep 21;23(23):e202200295. doi: 10.1002/cphc.202200295 (PMC10087976; doi:10.1002/cphc.202200295)
Supplement: Supplementary file 1 — Supporting Information [file CPHC-23-0-s001.pdf]

ChemPhysChem

Supporting Information

Resonances at Fundamental and Harmonic Frequencies for Selective Imaging of Sine-Wave Illuminated Reversibly Photoactivatable Labels

Agnès Pellissier-Tanon[+], Raja Chouket[+], Ruikang Zhang, Aliénor Lahlou, Agathe Espagne, Annie Lemarchand,* Vincent Croquette,* Ludovic Jullien,* and Thomas Le Saux*

Contents

S1 HIGHLIGHT theory 3
S1.1 Kinetic analysis of the two-state model . . . 3
S1.1.1 A two-state model with external sine-wave forcing . . . 3
S1.1.2 Single modulation at the wavelength λ_1 case (i1) - or λ_2 case (i2) . . . 4
Analytical derivation of the amplitudes of concentration oscillation . . . 4
Numerical derivation of the amplitudes of concentration oscillation . . . 6
S1.1.3 Two modulations in antiphase - case (ii) . . . 8
Analytical expression of the amplitudes of concentration oscillation . . . 8
Numerical derivation of the amplitudes of concentration oscillation . . . 9
S1.2 Evolution of fluorescence intensity . . . 12
S1.2.1 Single modulation at the wavelength λ_1 case (i1) . . . 13
S1.2.2 Single modulation at the wavelength λ_2 case (i2) . . . 14
S1.2.3 Two modulations in antiphase - case (ii) . . . 14
S1.3 Application to the discrimination of reversibly photoswitchable species . . . 17

S2 Experimental Section 23
S2.1 Protein production and purification . . . 23
S2.2 Mammalian cell culture and transfection . . . 23
S2.3 Reagents and solutions . . . 24
S2.4 Microfluidic devices . . . 24
S2.5 Epifluorescence setup for multiplexed imaging . . . 24
S2.6 Epifluorescence setup with inhomogeneous illumination . . . 24
S2.6.1 Materials . . . 24
S2.6.2 Scheme . . . 26
S2.6.3 Inhomogeneous illumination . . . 26
Model of light profile . . . 26
Calculation of the Pre-HIGHLIGHT and HIGHLIGHT-n signals for the model of inhomogeneous light profile . . . 28
S2.6.4 Characterization of the illumination profile . . . 29
S2.6.5 Samples for confocality validation . . . 29
S2.7 Generation of pure sine-wave light modulation . . . 29
S2.8 Video acquisition . . . 31
S2.9 Calibration of light intensities . . . 32
S2.10 Matlab code for extracting the HIGHLIGHT signals . . . 33
S2.11 Cell imaging . . . 35
S2.12 Confocal experiments . . . 35
S2.13 Photochemical parameters of the RSFPs . . . 35
S2.14 Acquisition parameters used for the HIGHLIGHT experiments . . . 35
S2.15 Softwares . . . 36

S3 Supplementary Figures 37

References 43

Author Contribution 43

S1 HIGHLIGHT theory

S1.1 Kinetic analysis of the two-state model

S1.1.1 A two-state model with external sine-wave forcing

The dynamical behavior of a reversibly photoswitchable label is assumed to be reliably described by the two-state exchange

$$C_1 \underset{k_2}{\overset{k_1}{\rightleftharpoons}} C_2 \tag{S1}$$

where the state C_1 is photochemically converted into the state C_2 under illumination I_1 at the wavelength λ_1. The conversion of C_2 into C_1 occurs under illumination I_2 at the wavelength λ_2 or by a slower thermally-driven process which is neglected if I_2 does not vanish. Introducing a light modulation at the wavelength λ_i of mean value I_i^0 and large amplitude α_i of order 1 at the angular frequency ω according to

$$I_i = I_i^0\left[1 + \alpha_i \sin\left(\omega t\right)\right] \tag{S2}$$

and assuming that the photochemical rate constants are proportional to light intensity yields

$$k_i = k_i^0\left[1 + \alpha_i \sin\left(\omega t\right)\right] \tag{S3}$$

for $i = 1, 2$.

Two cases are considered. The case (i) associated with a single modulation corresponds to either case (i1) associated with the modulation of I_1, i.e. $\alpha_1 = \alpha$ and $\alpha_2 = 0$, or case (i2) associated with the modulation of I_2, i.e. $\alpha_1 = 0$ and $\alpha_2 = \alpha$. In the following we illustrate case (i1). By symmetry case (i2) is deduced from case (i1) by exchanging the intensities I_1 and I_2, i.e. the rate constants k_1 and k_2, and exchanging the concentrations C_1 and C_2. The case (ii) is associated with two modulations in antiphase, i.e. $\alpha_1 = \alpha$ and $\alpha_2 = -\alpha$.

The evolution of the concentration C_1 is given by

$$\frac{\mathrm{d}C_1}{\mathrm{d}t} = -\left(k_1 + k_2\right)C_1 + k_2 C_{tot} \tag{S4}$$

where the total concentration obeys

$$C_{tot} = C_1 + C_2 \tag{S5}$$

We define the dimensionless deviation

$$c = \frac{C_1^0 - C_1}{C_{tot}} \tag{S6}$$

to the steady-state $C_1^0 = C_{tot}/(1 + K^0)$ with

$$K^0 = \frac{k_1^0}{k_2^0}. \tag{S7}$$

Using Eqs.(S4,S6) we find in case (i1)

$$\tau^0 \frac{\mathrm{d}c}{\mathrm{d}t} = -c + \alpha\left[\frac{K^0}{(1+K^0)^2} - \frac{K^0}{1+K^0}c\right]\sin(\omega t) \tag{S8}$$

and in case (ii)

$$\tau^0 \frac{\mathrm{d}c}{\mathrm{d}t} = -c + \alpha\left[\frac{2K^0}{(1+K^0)^2} - \frac{K^0-1}{1+K^0}c\right]\sin(\omega t) \tag{S9}$$

Beyond the relaxation time

$$\tau^0 = \frac{1}{k_1^0 + k_2^0} \tag{S10}$$

the system enters into a permanent regime in which the deviation $C = cC_{\rm tot}$ to the steady state is periodic and expressed using a Fourier series

$$C = a_0 + \sum_{n=1}^{+\infty} \left[a_n \cos(n\omega t) + b_n \sin(n\omega t)\right] \tag{S11}$$

where a_0 is the mean value of c and a_n and b_n designate the quadrature and in-phase amplitudes of concentration oscillation of the n-th harmonics.

In the following, we exploit a derivation called "harmonic balance" in mathematics. At steady-state, an ordinary differential equation admits a periodic solution, which is adopted as a truncated Fourier series. Then, balancing of harmonics yields an algebraic equation system in Fourier coefficients. Hence, we substitute for c from Eq.(S11) into Eq.(S8). Then we cancel the terms in front of the sine and cosine at each order n in order to retrieve linear equations, which are solved in order to retrieve the expressions of the a_n and b_n terms.

S1.1.2 Single modulation at the wavelength λ_1 case (i1) - or λ_2 case (i2)

Analytical derivation of the amplitudes of concentration oscillation Implementing harmonic balance, we substitute for c from Eq.(S11) into Eq.(S8), cancel the terms in front of the sine and cosine at each order n, and find the following linear equations

$$a_0 = -\frac{\alpha K^0}{2(1+K^0)} b_1 \tag{S12}$$

$$a_1\theta = b_1 - \frac{\alpha K^0 C_{\rm tot}}{(1+K^0)^2} - \frac{\alpha K^0}{2(1+K^0)}(a_2 - 2a_0) \tag{S13}$$

$$b_1\theta = -a_1 - \frac{\alpha K^0}{2(1+K^0)} b_2 \tag{S14}$$

$$a_n n\theta = b_n - \frac{\alpha K^0}{2(1+K^0)}(a_{n+1} - a_{n-1}) \quad \text{for} \quad n > 1 \tag{S15}$$

$$b_n n\theta = -a_n - \frac{\alpha K^0}{2(1+K^0)}(b_{n+1} - b_{n-1}) \quad \text{for} \quad n > 1 \tag{S16}$$

where

$$\theta = \omega\tau^0 \tag{S17}$$

The Fourier amplitudes a_n and b_n scale as α^n for $n \geq 1$. At leading order in α, the recurrence equations given in Eqs.(S12-S16) yield

$$a_0 \simeq -\alpha \frac{K^0}{2(1+K^0)} b_1 \tag{S18}$$

$$a_1 \simeq -\alpha \frac{K^0}{(1+K^0)^2} \frac{\theta}{1+\theta^2} C_{\rm tot} \tag{S19}$$

$$b_1 \simeq \alpha \frac{K^0}{(1+K^0)^2} \frac{1}{1+\theta^2} C_{\rm tot} \tag{S20}$$

$$a_n \simeq \alpha \frac{K^0}{2(1+K^0)(1+n^2\theta^2)} \left(n\theta a_{n-1} + b_{n-1}\right) \quad \text{for} \quad n > 1 \tag{S21}$$

$$b_n \simeq -\alpha \frac{K^0}{2(1+K^0)(1+n^2\theta^2)} \left(a_{n-1} - n\theta b_{n-1}\right) \quad \text{for} \quad n > 1 \tag{S22}$$

The explicit expressions of the amplitudes of concentration oscillation up to the fourth order are

$$a_0 \simeq -\alpha^2 \frac{(K^0)^2}{2(1+K^0)^3} \frac{1}{1+\theta^2} C_{\rm tot} \tag{S23}$$

$$a_2 \simeq \alpha^2 \frac{(K^0)^2}{2(1+K^0)^3} \frac{1-2\theta^2}{(1+\theta^2)(1+4\theta^2)} C_{\rm tot} \tag{S24}$$

$$b_2 \simeq \alpha^2 \frac{(K^0)^2}{2(1+K^0)^3} \frac{3\theta}{(1+\theta^2)(1+4\theta^2)} C_{\rm tot} \tag{S25}$$

$$a_3 \simeq \alpha^3 \frac{(K^0)^3}{2^2(1+K^0)^4} \frac{6\theta(1-\theta^2)}{(1+\theta^2)(1+4\theta^2)(1+9\theta^2)} C_{\rm tot} \tag{S26}$$

$$b_3 \simeq -\alpha^3 \frac{(K^0)^3}{2^2(1+K^0)^4} \frac{1-11\theta^2}{(1+\theta^2)(1+4\theta^2)(1+9\theta^2)} C_{\rm tot} \tag{S27}$$

$$a_4 \simeq -\alpha^4 \frac{(K^0)^4}{2^3(1+K^0)^5} \frac{1-35\theta+24\theta^2}{(1+\theta^2)(1+4\theta^2)(1+9\theta^2)(1+16\theta^2)} C_{\rm tot} \tag{S28}$$

$$b_4 \simeq -\alpha^4 \frac{(K^0)^4}{2^3(1+K^0)^5} \frac{10\theta(1-5\theta^2)}{(1+\theta^2)(1+4\theta^2)(1+9\theta^2)(1+16\theta^2)} C_{\rm tot} \tag{S29}$$

with a_1 and b_1 given in Eqs.(S19,S20).

The amplitudes of concentration oscillation depend on two parameters, the equilibrium constant K^0 and the scaled angular frequency θ. Assuming that, for a given order $n-1$, we have

$$a_{n-1} = \alpha^{n-1} \frac{(K^0)^{n-1}}{2^{n-1}(1+K^0)^n} \frac{f_{n-1}(\theta)}{\Pi_{k=1}^{n-1}(1+k^2\theta^2)} C_{\rm tot} \tag{S30}$$

$$b_{n-1} = \alpha^{n-1} \frac{(K^0)^{n-1}}{2^{n-1}(1+K^0)^n} \frac{g_{n-1}(\theta)}{\Pi_{k=1}^{n-1}(1+k^2\theta^2)} C_{\rm tot} \tag{S31}$$

where $f_{n-1}(\theta)$ and $g_{n-1}(\theta)$ are polynomial functions independent of K^0 , we use Eqs.(S21,S22) to show that Eqs.(S30,S31) are valid at order n and consequently at all order. In order to determine the extrema of the amplitudes of concentration oscillation, we first differentiate Eqs.(S30,S31) with respect to K^0 and obtain the resonant value $K_n^{0R} = n$ for the equilibrium constant at order n. The polynomial functions $f_{2k+1}(\theta)$ associated with odd orders and $g_{2k}(\theta)$ associated with even orders vanish for $\theta = 0$ contrary to $f_{2k}(\theta)$ and $g_{2k+1}(\theta)$. The amplitudes of concentration oscillation a_{2k+1} and b_{2k} display a pronounced extremum contrary to a_{2k} and b_{2k+1} which behave as a low-pass filter with respect to θ. The resonant value θ_n^R of the scaled angular frequency is close to $1/(2n-1)$.

Numerical derivation of the amplitudes of concentration oscillation Equations (S12-S16) involve an infinite number of Fourier amplitudes and their numerical solution requires a truncation. We studied the convergence of the solution of the four first orders using truncation at increasing values of n. Considering the Fourier amplitudes up to $n = 5$ is enough to observe the desired convergence. The resonances shown in Fig. S1 are associated with the values given in Tab. S1.

The amplitudes of concentration oscillation with extrema do not have the same sign, a_1 and b_4 being negative and b_2 and a_3 positive. The greater the order, the less symmetric the resonance peak and the smaller its width at half maximum. For a large modulation of light excitation $\alpha = 1$, the value of the amplitudes at the resonance decrease by less than a factor of 2 as the order n increases. The resonant values K_n^{0R} of the equilibrium constant increase with the order n whereas the resonant values θ_n^R of the scaled angular frequency decrease. The analytical expressions underestimate the peak displacement as the order changes and they overestimate the decrease of the amplitude at the resonance and the width at half maximum.

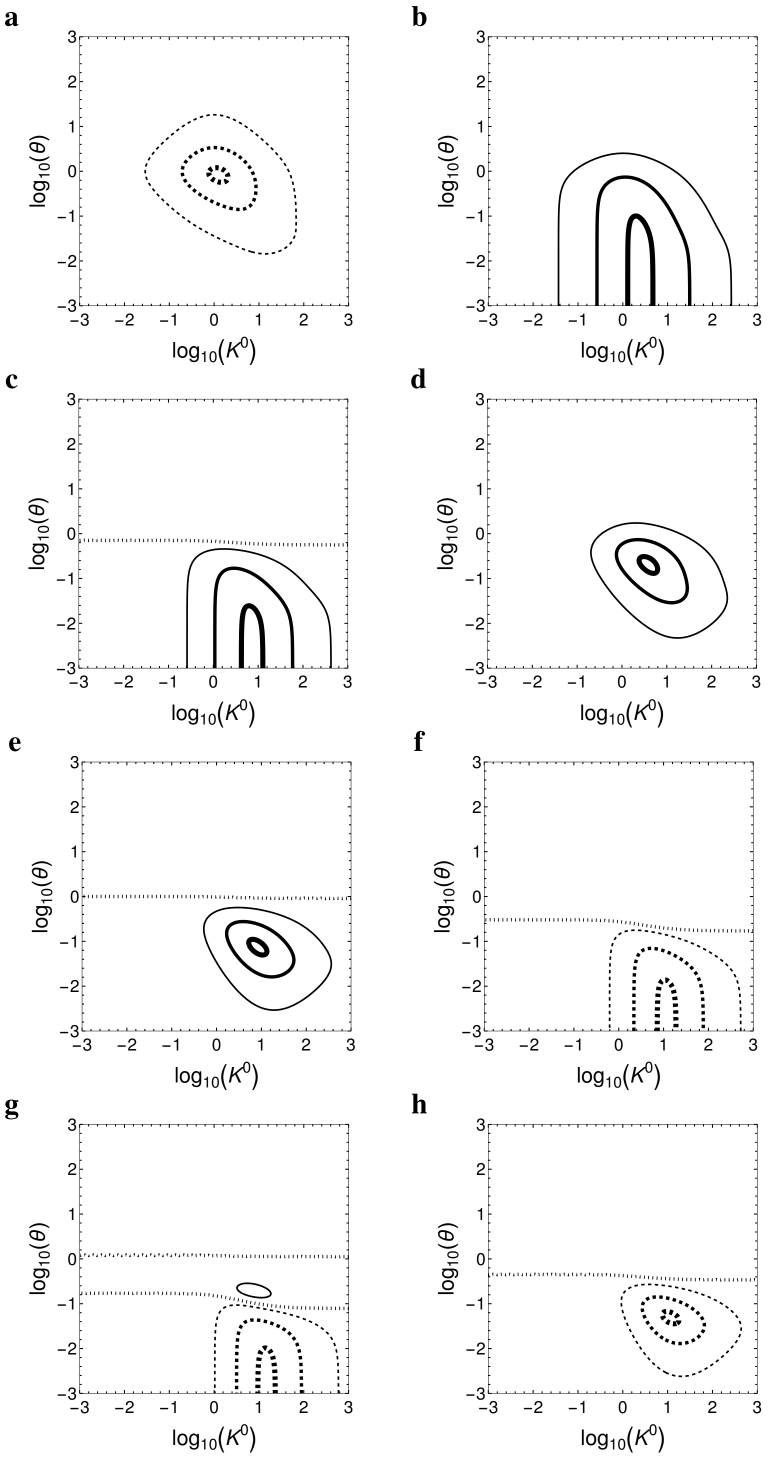

Figure S1: Amplitudes of concentration oscillation driven by single modulation at the wavelength λ_1 (case (i1)) or λ_2 (case (i2)) and of fluorescence oscillation driven by single modulation at the wavelength λ_2 (case (i2)) deduced from the numerical solutions of Eqs.(S12-S16) truncated at the fifth order for $\alpha = 1$. (a): a_1, (b): b_1, (c): a_2, (d): b_2, (e): a_3, (f): b_3, (g): a_4, (h): b_4. The parameter values for which the amplitudes are equal to 0 are represented by a dotted line. The other contour lines are set at 95 % (thick lines), 50 % (medium lines), and 10 % (thin lines) of the extremum value reached in the represented parameter domain, the positive values being associated with a plain line and the negative values with a dashed line.

S1.1.3 Two modulations in antiphase - case (ii)

Analytical expression of the amplitudes of concentration oscillation Following the same method as in section S1.1.2 for a single modulation, we find the following linear equations for the amplitudes of concentration oscillation in the case of two modulations in antiphase

$$a_0 = -\frac{\alpha(K^0-1)}{2(1+K^0)}b_1 \tag{S32}$$

$$a_1\theta = b_1 - \frac{2\alpha K^0 C_{\rm tot}}{(1+K^0)^2} - \frac{\alpha(K^0-1)}{2(1+K^0)}(a_2-2a_0) \tag{S33}$$

$$b_1\theta = -a_1 - \frac{\alpha(K^0-1)}{2(1+K^0)}b_2 \tag{S34}$$

$$a_n n\theta = b_n - \frac{\alpha(K^0-1)}{2(1+K^0)}(a_{n+1}-a_{n-1}) \quad \text{for} \quad n>1 \tag{S35}$$

$$b_n n\theta = -a_n - \frac{\alpha(K^0-1)}{2(1+K^0)}(b_{n+1}-b_{n-1}) \quad \text{for} \quad n>1 \tag{S36}$$

The explicit expressions of the amplitudes of concentration oscillation up to the fourth order are

$$a_0 \simeq -\alpha^2\frac{K^0(K^0-1)}{2(1+K^0)^3}\frac{1}{1+\theta^2}C_{\rm tot} \tag{S37}$$

$$a_1 \simeq -2\alpha\frac{K^0}{(1+K^0)^2}\frac{\theta}{1+\theta^2}C_{\rm tot} \tag{S38}$$

$$b_1 \simeq 2\alpha\frac{K^0}{(1+K^0)^2}\frac{1}{1+\theta^2}C_{\rm tot} \tag{S39}$$

$$a_2 \simeq \alpha^2\frac{K^0(K^0-1)}{2(1+K^0)^3}\frac{1-2\theta^2}{(1+\theta^2)(1+4\theta^2)}C_{\rm tot} \tag{S40}$$

$$b_2 \simeq \alpha^2\frac{K^0(K^0-1)}{2(1+K^0)^3}\frac{3\theta}{(1+\theta^2)(1+4\theta^2)}C_{\rm tot} \tag{S41}$$

$$a_3 \simeq \alpha^3\frac{K^0(K^0-1)^2}{2^2(1+K^0)^4}\frac{6\theta(1-\theta^2)}{(1+\theta^2)(1+4\theta^2)(1+9\theta^2)}C_{\rm tot} \tag{S42}$$

$$b_3 \simeq -\alpha^3\frac{K^0(K^0-1)^2}{2^2(1+K^0)^4}\frac{1-11\theta^2}{(1+\theta^2)(1+4\theta^2)(1+9\theta^2)}C_{\rm tot} \tag{S43}$$

$$a_4 \simeq -\alpha^4\frac{K^0(K^0-1)^3}{2^3(1+K^0)^5}\frac{1-35\theta+24\theta^2}{(1+\theta^2)(1+4\theta^2)(1+9\theta^2)(1+16\theta^2)}C_{\rm tot} \tag{S44}$$

$$b_4 \simeq -\alpha^4\frac{K^0(K^0-1)^3}{2^3(1+K^0)^5}\frac{10\theta(1-5\theta^2)}{(1+\theta^2)(1+4\theta^2)(1+9\theta^2)(1+16\theta^2)}C_{\rm tot} \tag{S45}$$

The amplitudes of concentration oscillation a_1 and b_1 at the first order given in Eq.(S38,S39) driven by two modulations in antiphase are twice the amplitudes of concentration oscillation a_1 and b_1 given in Eq.(S19,S20) driven by a single modulation.

Table S1: Amplitudes R of concentration oscillation with extrema driven by single modulation at the wavelength λ_1 (case (i1)) or at the wavelength λ_2 (case (i2)) and of fluorescence oscillation driven by single modulation at the wavelength λ_2 (case (i2)), resonant parameter values K_n^{0R} and θ_n^R up to the fourth order deduced from the numerical solutions of Eqs.(S12-S16) truncated at the fifth order for $\alpha = 1$.

Order	R	K_n^{0R}	θ_n^R
1	a_1^R	1.24	0.84
2	b_2^R	3.83	0.21
3	a_3^R	8.25	0.07
4	b_4^R	11.55	0.05

Using a similar mathematical induction as in case (i1), we show that for all orders n the amplitudes obey

$$a_n = \alpha^n \frac{K^0(K^0-1)^{n-1}}{2^n(1+K^0)^{n+1}} \frac{f_n(\theta)}{\Pi_{k=1}^n (1+k^2\theta^2)} C_{\text{tot}} \quad \text{(S46)}$$

$$b_n = \alpha^n \frac{K^0(K^0-1)^{n-1}}{2^n(1+K^0)^{n+1}} \frac{g_n(\theta)}{\Pi_{k=1}^n (1+k^2\theta^2)} C_{\text{tot}} \quad \text{(S47)}$$

where $f_n(\theta)$ and $g_n(\theta)$ are the same polynomial functions independent of K^0 as in Eqs.(S30,S31) leading in particular to the same behavior in θ. The resonant value of the equilibrium constant $K_n^{0R} = n \pm \sqrt{n^2-1}$ at order n is deduced from Eqs.(S46,S47). The amplitude a_1 displays a single extremum of coordinates (K_1^{0R}, θ_1^R). From the second order the amplitudes a_{2k+1} and b_{2k} possess two extrema $(K_n^{0R_1}, \theta_n^R)$ and $(K_n^{0R_2}, \theta_n^R)$ associated with the same resonant value of θ identical to the value obtained in case (i1). The amplitudes a_{2k} and b_{2k+1} behave as a low-pass filter with respect to θ.

Numerical derivation of the amplitudes of concentration oscillation Figure S2 displays the amplitudes of concentration oscillation in the (K^0, θ) space obtained using a numerical solution of Eqs.(S32-S36) truncated at the fifth order. Tab. S2 gives the amplitudes R with extrema and the resonant parameter values deduced from the numerical approach.

The amplitudes of odd order display a mirror symmetry with respect to the $K^0 = 1$-plane in the $(\log_{10}(K^0), \log_{10}(\theta))$ space. The amplitudes of even order display a mirror antisymmetry with respect to the $K^0 = 1$-plane. The greater the order, the smaller the width of the resonance peak at half maximum. For a large modulation of light excitation $\alpha = 1$, the absolute amplitudes at the extremum decrease by less than a factor of 2 when the order n increases by 1 from $n = 2$. A more severe decrease of about a factor of 3 is observed when switching from $n = 1$ to $n = 2$.

The resonant values in K^0 obey $K_n^{0R_1} < K_1^{0R} < K_n^{0R_2}$ for $n > 1$. The values $K_n^{0R_1}$ and θ_n^R decrease as the order n increases. On the contrary $K_n^{0R_2}$ increases as n increases. The analytical expressions underestimate the resonance displacement as the order changes and they overestimate the decrease of the amplitude at the extremum and the width at half maximum of the peak.

Table S2: Amplitudes R of concentration oscillation with extrema driven by two modulations in antiphase (case (ii)), resonant parameter values K_1^{0R} and θ_1^R, $K_n^{0R_1}$, $K_n^{0R_2}$ and $\theta_n^{R_1} = \theta_n^{R_2}$ up to the fourth order deduced from the numerical solutions of Eqs.(S32-S36) truncated at the fifth order for $\alpha = 1$.

Order	R	K_1^{0R}		θ_1^R
1	a_1^R	1.00		1.00
Order	R	$K_n^{0R_1}$	$K_n^{0R_2}$	$\theta_n^{R_1} = \theta_n^{R_2}$
2	$b_2^{R_1}$, $b_2^{R_2}$	0.15	6.60	0.24
3	$a_3^{R_1}$, $a_3^{R_2}$	0.07	15.04	0.08
4	$b_4^{R_1}$, $b_4^{R_2}$	0.04	22.36	0.05

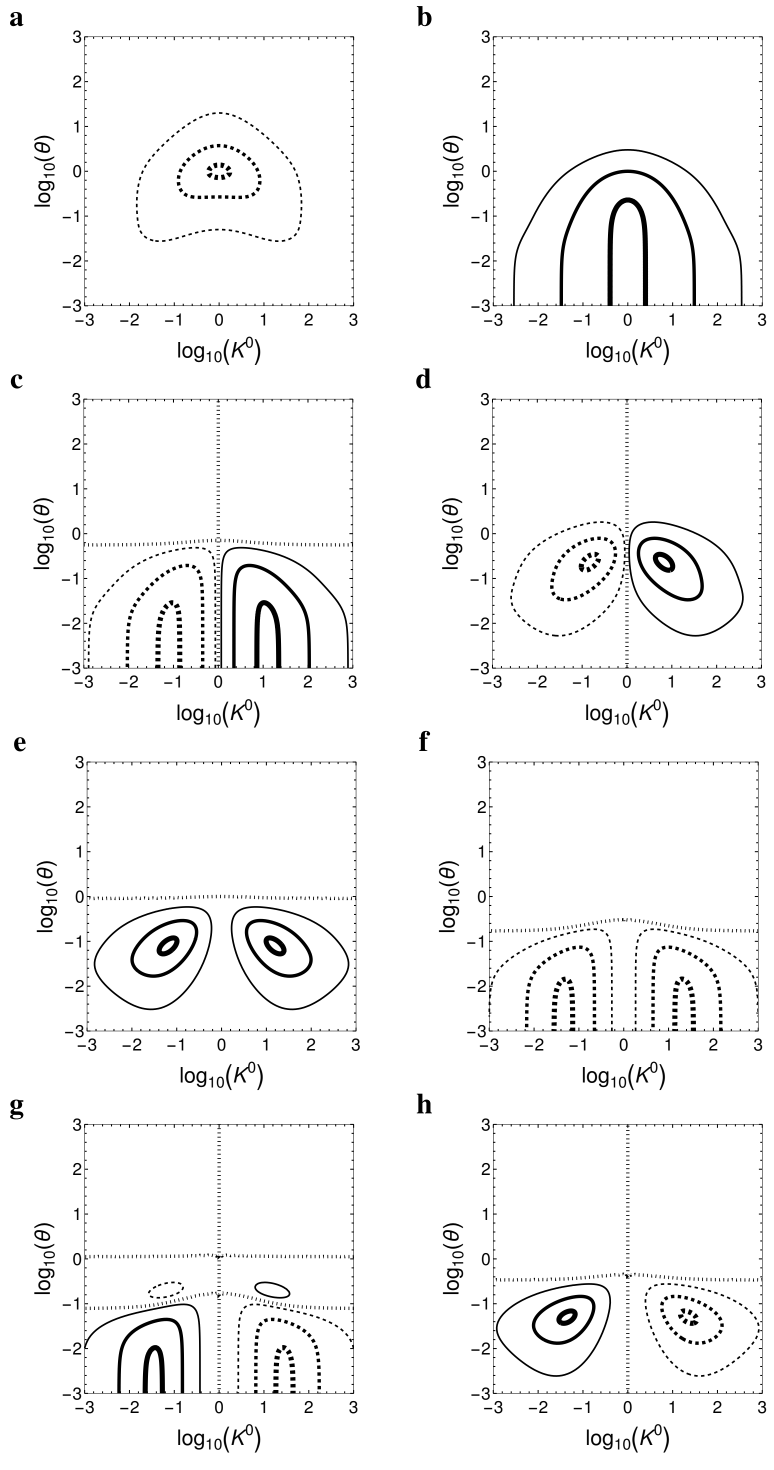

Figure S2: Amplitudes of concentration oscillation driven by two modulations in antiphase (case (ii)) deduced from the numerical solutions of Eqs.(S32-S36) truncated at the fifth order for $\alpha = 1$. (a): a_1, (b): b_1, (c): a_2, (d): b_2, (e): a_3, (f): b_3, (g): a_4, (h): b_4. The parameter values for which the amplitudes are equal to 0 are represented by a dotted line. The other contour lines are set at 95 % (thick lines), 50 % (medium lines), and 10 % (thin lines) of the extremum value reached in the represented parameter domain, the positive values being associated with a plain line and the negative values with a dashed line.

Figure S3 displays the variation of the amplitudes R of concentration oscillation with extrema, the surface S at half

maximum of the peak in the $(\log_{10}(K^0), \log_{10}(\theta))$ space, and the ratio R/S with respect to the order n for case (i1) (single modulation) and case (ii) (two modulations in antiphase). The decrease of the surface S shows the increase of the resonance sharpness as the order increases. Nevertheless the decrease of the amplitude R is faster than the decrease of the surface S. However the ratio R/S reaches a plateau at large n. A balance has to be found between signal strength and resonance sharpness. The analytical results underestimate the amplitudes at the extremum and the surfaces at half maximum. Except for the first order, for which the amplitude associated with case (ii) is much larger than the amplitude of case (i1), both cases lead to close resonance properties.

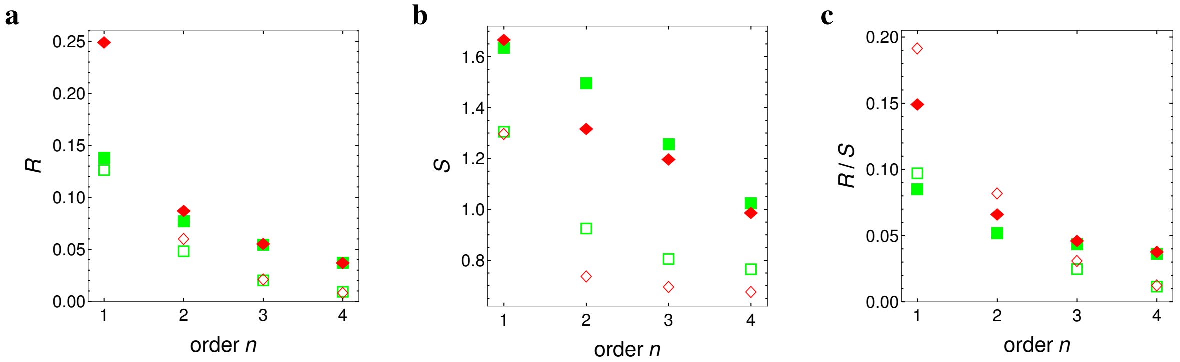

Figure S3: (a) Absolute amplitudes R of concentration oscillation at the extremum defined in Tabs. S1 and S2, (b) surface S at half maximum of the peak in the $(\log_{10}(K^0), \log_{10}(\theta))$ space, and (c) ratio R/S versus order n for $\alpha = 1$. The green squares correspond to a single modulation associated with case (i1), the red diamonds to two modulations in antiphase associated with case (ii), the open symbols to the analytical results, and the solid symbols to the numerical results. Total concentration $C_{\text{tot}} = 1$.

S1.2 Evolution of fluorescence intensity

In this section we address the case where the observable is fluorescence intensity. For the sake of simplicity, we consider a single significant brightness Q for a single species C_i for i equals 1 or 2 at the wavelength λ_1. We introduce the scaled fluorescence intensity

$$I_{\text{F}} = \frac{I_1 C_i}{I_1^0 C_{tot}} \tag{S48}$$

where $i = 1$ for negative photoswitchable fluorophores and $i = 2$ for positive photoswitchable fluorophores. Whereas C_1 is fluorescent for negative photoswitchable fluorophores, C_2 is fluorescent for positive ones. Contrary to the concentration the fluorescence intensity is not symmetrical with respect to the exchange of the light intensities I_1 and I_2.

The fluorescence intensity is expressed using a Fourier series

$$I_{\text{F}} = I_{\text{F}}^0 + \sum_{n=1}^{+\infty} \left[A_n \cos(n\omega t) + B_n \sin(n\omega t)\right] \tag{S49}$$

where I_{F}^0 is the mean fluorescence intensity and A_n and B_n designate the quadrature and in-phase amplitudes of fluorescence oscillation of the n-th harmonics.

Using Eqs.(S6,S48) we obtain

$$I_{\text{F}} = \frac{I_1 \left(C_1^0 - C\right)}{I_1^0} \tag{S50}$$

for negative photoswitchers and

$$I_{\mathrm{F}} = \frac{I_1 \left(C_{tot} - C_1^0 + C\right)}{I_1^0} \tag{S51}$$

for positive photoswitchers.

Substituting for I_{F} from Eq.(S49), I_1 from Eq.(S2), and c from Eq.(S11) into Eq.(S50), we cancel the terms in front of the sine and cosine at each order n and find the following linear equations

$$I_{\mathrm{F}}^0 = \frac{C_{\mathrm{tot}}}{1+K^0} - a_0 - \frac{\alpha_1}{2} b_1 \tag{S52}$$

$$A_1 = -a_1 - \frac{\alpha_1}{2} b_2 \tag{S53}$$

$$B_1 = -b_1 + \alpha_1 \left(\frac{C_{\mathrm{tot}}}{1+K^0} - a_0 + \frac{1}{2} a_2\right) \tag{S54}$$

$$A_n = -a_n + \frac{\alpha_1}{2} \left(b_{n-1} - b_{n+1}\right) \tag{S55}$$

$$B_n = -b_n - \frac{\alpha_1}{2} \left(a_{n-1} - a_{n+1}\right) \tag{S56}$$

for negative photoswitchers. Substituting for I_{F} from Eq.(S49), I_1 from Eq.(S2), and c from Eq.(S11) into Eq.(S51), we obtain

$$I_{\mathrm{F}}^0 = \frac{K^0 C_{\mathrm{tot}}}{1+K^0} + a_0 + \frac{\alpha_1}{2} b_1 \tag{S57}$$

$$A_1 = a_1 + \frac{\alpha_1}{2} b_2 \tag{S58}$$

$$B_1 = b_1 + \alpha_1 \left(\frac{K^0 C_{\mathrm{tot}}}{1+K^0} + a_0 - \frac{1}{2} a_2\right) \tag{S59}$$

$$A_n = a_n - \frac{\alpha_1}{2} \left(b_{n-1} - b_{n+1}\right) \tag{S60}$$

$$B_n = b_n + \frac{\alpha_1}{2} \left(a_{n-1} - a_{n+1}\right) \tag{S61}$$

for positive photoswitchers. Except for I_{F}^0 and B_1, the amplitudes of fluorescence oscillation for positive and negative photoswitchers only differ by their sign. When the light intensity I_1 at the wavelength λ_1 is not modulated, i.e. in case (i2) with $\alpha_1 = 0$ and $\alpha_2 = \alpha$, the amplitudes of fluorescence oscillation, A_n and B_n, are equal to the amplitudes a_n and b_n of concentration oscillations. When the light intensity I_1 at the wavelength λ_1 is modulated, i.e. in case (i1) with $\alpha_1 = \alpha$ and $\alpha_2 = 0$ and in case (ii) with $\alpha_1 = \alpha$ and $\alpha_2 = -\alpha$, the amplitudes A_n and B_n are linear combination of amplitudes a_n and b_n either with or without extremum.

S1.2.1 Single modulation at the wavelength λ_1 case (i1)

The case (i1) is associated with a single modulation of light intensity I_1 at the wavelength λ_1 i.e. $\alpha_1 = \alpha$, $\alpha_2 = 0$. Substituting for the amplitudes of concentration oscillation from Eqs.(S19,S20,S23-S29) into Eqs.(S52-S61) for the Fourier amplitudes A_n and B_n, we obtain approximate analytical expressions for the amplitudes of fluorescence oscillation. Using Eqs.(S57-S59) and numerical solutions of the amplitudes of concentration oscillation a_n and b_n after truncation of Eqs.(S12-S16) at the fifth order, we obtain numerical solutions of the amplitudes of fluorescence oscillation. The numerical results are given in Fig. S4. The first-order amplitude A_1 is very close to a_1 showing a single resonance denoted by the exponent R. From the second order, the odd quadrature amplitudes A_{2k+1} and even in-phase amplitudes B_{2k} have resonances but display a richer behavior than the corresponding amplitudes a_{2k+1} and b_{2k} of the concentrations. In particular they present two peaks associated with opposite signs, implying that the amplitudes vanish on a specific line in the

(K^0, θ) space. The resonance associated with the peak of larger amplitude in absolute value is denoted by an exponent R_1 and the smaller peak with R_2. Table S3 gives the characteristics of the resonances. Due to the presence of two peaks the amplitudes of fluorescence oscillation are smaller than the corresponding amplitudes of the concentrations.

Table S3: Amplitudes R of fluorescence oscillation with extrema driven by the single modulation at the wavelength λ_1 (case (i1)), resonant parameter values K_1^{0R} and θ_1^R, $K_n^{0R_1}$, $K_n^{0R_2}$, $\theta_n^{R_1}$, and $\theta_n^{R_2}$ up to the third order deduced from the numerical solutions of Eqs.(S12-S16) truncated at the fifth order and introduced in Eqs.(S57-S61) for positive photoswitchers and $\alpha = 1$.

Order	R	K_1^{0R}		θ_1^R	
1	A_1^R	1.08		1.00	
Order	R	$K_n^{0R_1}$	$K_n^{0R_2}$	$\theta_n^{R_1}$	$\theta_n^{R_2}$
2	$B_2^{R_1}$, $B_2^{R_2}$	0.88	5.16	1.33	0.18
3	$A_3^{R_1}$, $A_3^{R_2}$	2.22	12.21	0.50	0.05

S1.2.2 Single modulation at the wavelength λ_2 case (i2)

The case (i2) is associated with a single modulation of light intensity I_2 at the wavelength λ_2 i.e. $\alpha_1 = 0$, $\alpha_2 = \alpha$. The amplitudes of fluorescence oscillation, A_n and B_n, are equal to the amplitudes a_n and b_n of concentration oscillations. The results are given in Fig. S1 and Tab. S1 after exchanging the intensities I_1 and I_2 and the concentrations C_1 and C_2.

S1.2.3 Two modulations in antiphase - case (ii)

The case (ii) is associated with two antiphase modulations of light intensities I_1 at the wavelength λ_1 and I_2 at the wavelength λ_2, i.e. $\alpha_1 = \alpha$ and $\alpha_2 = -\alpha$. Following the same method as in section S1.2.1, we find the analytical and numerical expressions of the amplitudes A_n and B_n of fluorescence oscillation. Fig. S5 displays the numerical results. Unlike the amplitudes of concentration oscillation, the amplitudes of fluorescence oscillation do not have a mirror symmetry. At the first order the amplitude A_1 is similar to the concentration amplitude a_1. From the second order the odd quadrature amplitudes A_{2k+1} and even in-phase amplitudes B_{2k} display two resonances. Contrary to the amplitudes a_{2k+1} and b_{2k}, the two amplitudes A_{2k+1} and B_{2k} have always opposite signs and different absolute values. The amplitudes having two resonances vanish along a specific line in the (K^0, θ) space. The resonance associated with the peak of larger amplitude in absolute value is denoted by an exponent R_1 and the smaller peak by R_2. Table S4 gives the coordinates of the resonances.

Table S4: Amplitudes R of fluorescence oscillation with extrema driven by two modulations in antiphase (case (ii)), resonant parameter values K_1^{0R} and θ_1^R, $K_n^{0R_1}$, $K_n^{0R_2}$, $\theta_n^{R_1}$, and $\theta_n^{R_2}$ up to the third order deduced from the numerical solutions of Eqs.(S32-S36) truncated at the fifth order and introduced in Eqs.(S57-S61) for positive photoswitchers and $\alpha = 1$.

Order	R	K_1^{0R}		θ_1^R	
1	A_1^R	0.80		0.97	
Order	R	$K_n^{0R_1}$	$K_n^{0R_2}$	$\theta_n^{R_1}$	$\theta_n^{R_2}$
2	$B_2^{R_1}$, $B_2^{R_2}$	0.21	10.06	0.33	0.18
3	$A_3^{R_1}$, $A_3^{R_2}$	0.07	3.96	0.09	0.50

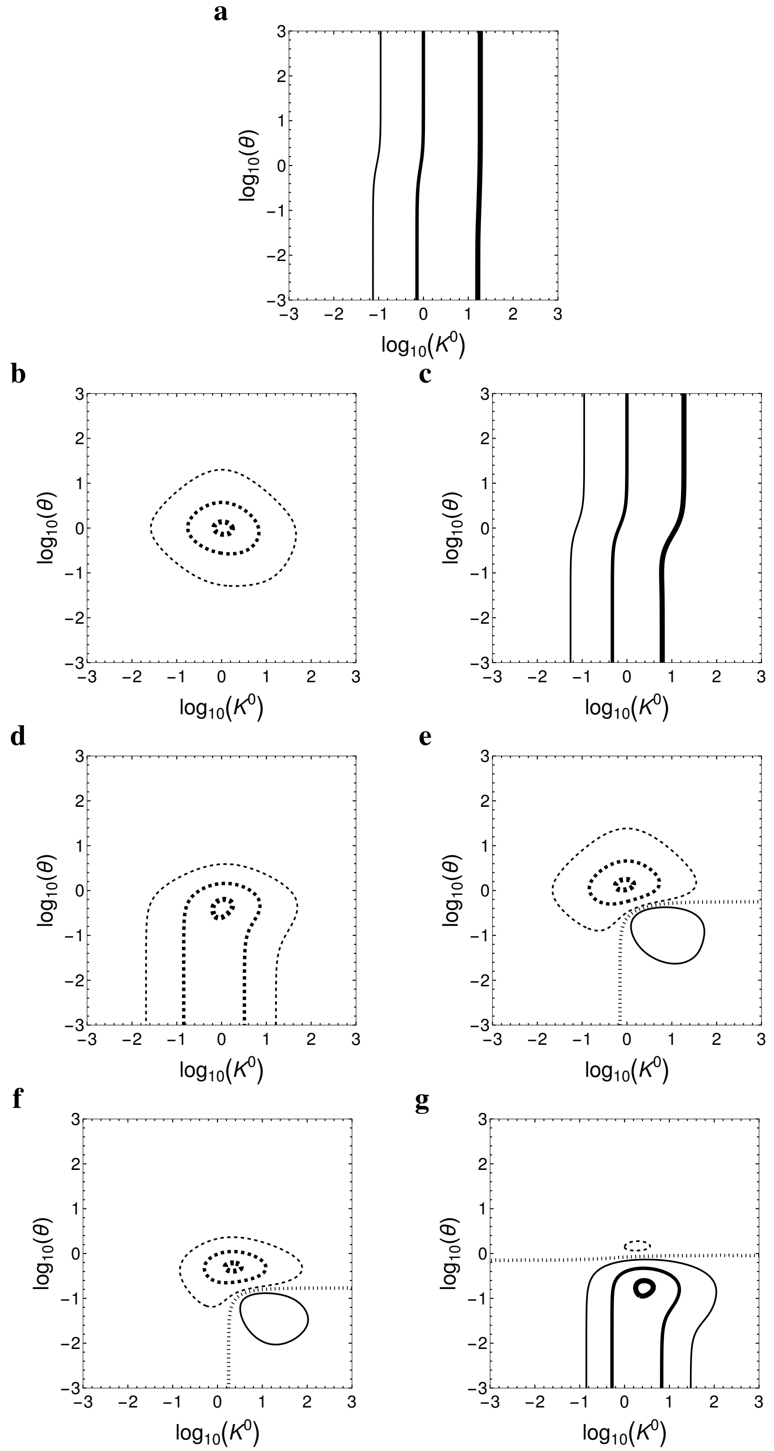

Figure S4: Amplitudes of fluorescence oscillation driven by single modulation at the wavelength λ_1 (case (i1)) deduced from the numerical solutions of Eqs.(S12-S16) truncated at the fifth order and introduced in Eqs.(S57-S61) for positive photoswitchers and $\alpha = 1$. (a): I_{F}^0, (b): A_1, (c): B_1, (d): A_2, (e): B_2, (f): A_3, (g): B_3. The parameter values for which the amplitudes are equal to 0 are represented by a dotted line. The other contour lines are set at 95 % (thick lines), 50 % (medium lines), and 10 % (thin lines) of the extremum value reached in the represented parameter domain, the positive values being associated with a plain line and the negative values with a dashed line.

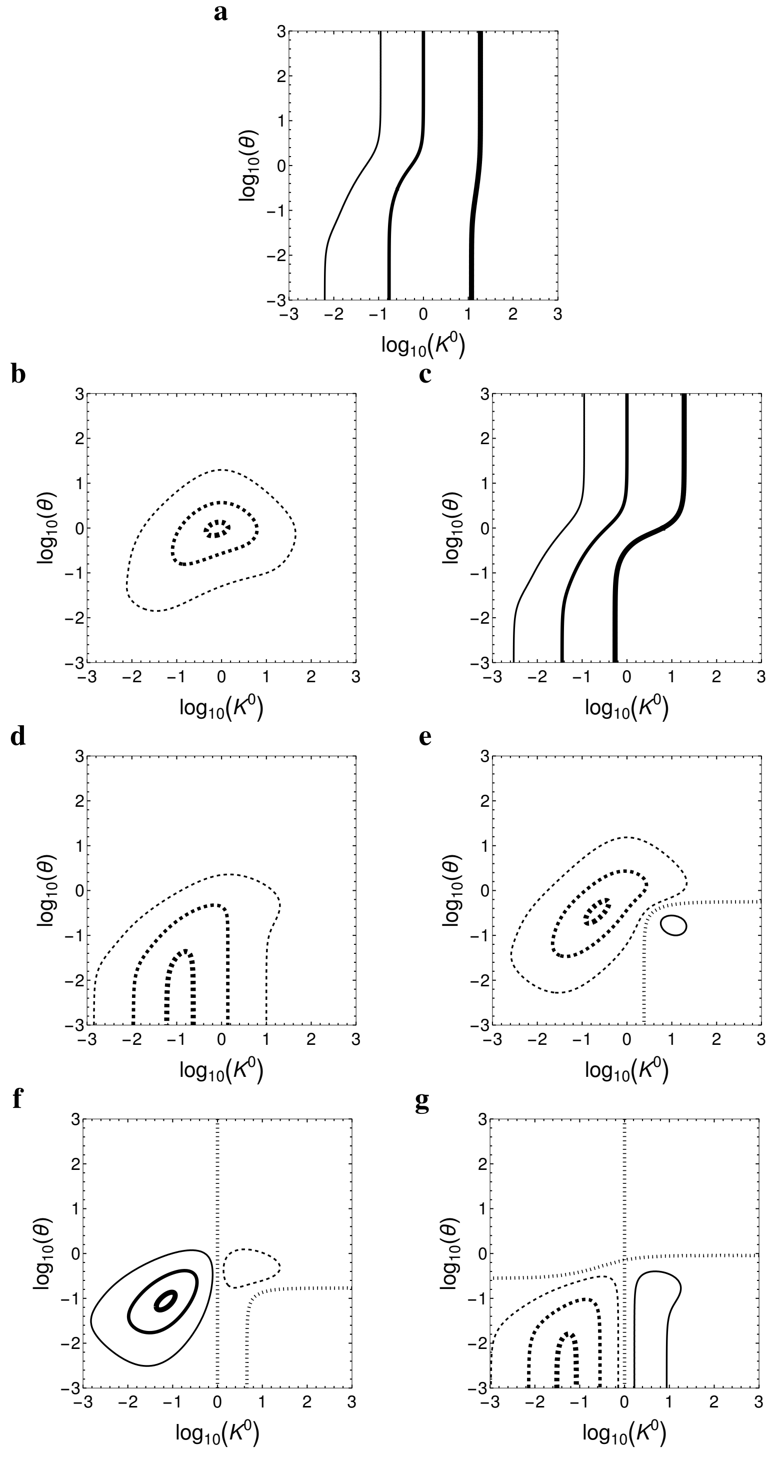

Figure S5: Amplitudes of fluorescence oscillation driven by two modulations in antiphase (case (ii)) deduced from the numerical solutions of Eqs.(S32-S36) truncated at the fifth order and introduced in Eqs.(S57-S61) for positive photo-switchers and $\alpha = 1$. (a): $I_{\rm F}^0$, (b): A_1, (c): B_1, (d): A_2, (e): B_2, (f): A_3, (g): B_3. The parameter values for which the amplitudes are equal to 0 are represented by a dotted line. The other contour lines are set at 95 % (thick lines), 50 % (medium lines), and 10 % (thin lines) of the extremum value reached in the represented parameter domain, the positive values being associated with a plain line and the negative values with a dashed line.

Figure S6 displays the variation of the amplitudes R of fluorescence oscillation with extrema, the surface S at half

maximum of the peaks in the $(\log_{10}(K^0), \log_{10}(\theta))$ space, and the ratio R/S with respect to the order n for case (i1) (single modulation at λ_1), case (i2) (single modulation at λ_2), and case (ii) (two modulations in antiphase). The analytical results underestimate the amplitudes at the extremum and the surfaces at half maximum. Regardless of the order n, the resonance R_1 of larger amplitude is significantly greater for two modulations than for a single modulation, case (i2) being slightly more favorable than case (i1). Contrary to the resonance R_1, the resonance R_2 of smaller amplitude does not sensitively decrease as the order increases from $n = 2$. The surface at half width S is anomalously large in the case (ii) for $n = 2$. Interestingly for case (i1) and resonance R_1, the decrease of S as n increases is more marked than in the other cases. The ratio R/S of the amplitude at the extremum and the surface at half width accounts for the sharpness of the resonance and remains nearly constant in all cases when switching from $n = 2$ to $n = 3$.

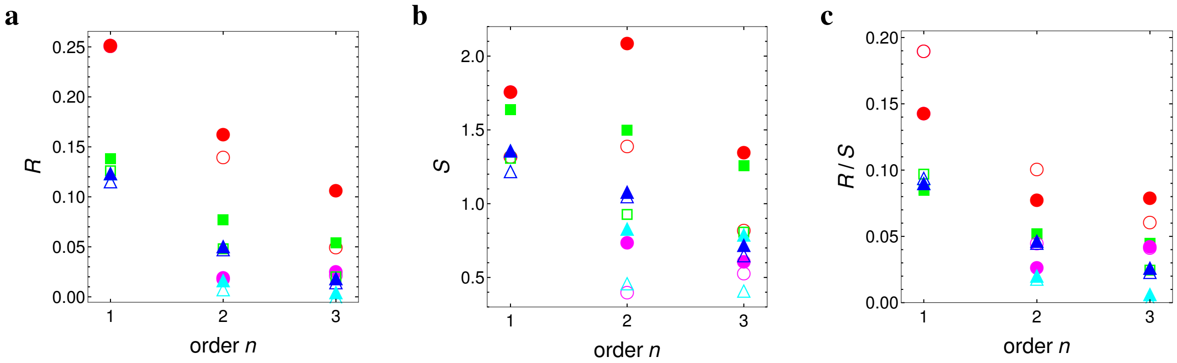

Figure S6: (a) Absolute amplitudes R of fluorescence oscillation at the extremum defined in Tabs. S1, S3, and S4, (b) surface S at half maximum of the peaks in the $(\log_{10}(K^0), \log_{10}(\theta))$ space, and (c) ratio R/S versus order n for $\alpha = 1$. The green squares correspond to the resonance R associated with a single modulation and case (i2). The blue and cyan triangles correspond to a single modulation associated with case (i1) and the red and magenta circles to two modulations in antiphase associated with case (ii). The blue triangles and the red circles correspond to the resonance R_1 of larger amplitude. The cyan triangles and the magenta circles correspond to the resonance R_2 of smaller amplitude. The open and solid symbols are associated with the analytical and numerical results, respectively. Total concentration $C_{\text{tot}} = 1$.

S1.3 Application to the discrimination of reversibly photoswitchable species

As shown in Eqs.(S8,S9,S50,S51), the analysis of the resonant behaviors of the amplitudes of concentration or fluorescence oscillation reveals the minimal number of dimensionless independent parameters. However the discrimination of several reversibly photoswitchable species requires switching from the two dimensionless parameters, K^0 and θ, to two experimentally relevant control parameters chosen among the mean light intensities I_1^0 and I_2^0 and the angular frequency ω.

The mean value of the photochemical rate constant of the forward reaction given in Eq.(S1) is assumed to be given by

$$k_1^0 = \sigma_1 I_1^0 \tag{S62}$$

where σ_1 is the cross section and I_1^0, the mean light intensity at the wavelength λ_1. For a single modulation at the wavelength λ_1 (case (i1)) two subcases are considered depending on the values of the mean light intensity I_2^0 at the wavelength λ_2. Case (i1a) corresponds to a vanishing light intensity I_2^0 leading to a thermal rate constant

$$k_2^0 = k^\Delta \tag{S63}$$

The properties of the photochemical system are then set by the two control parameters I_1^0 and ω. Case (i1b) corresponds

to a sufficiently large light intensity I_2^0 such that

$$\sigma_2 I_2^0 \gg k^\Delta \tag{S64}$$

leading to

$$k_2^0 = \sigma_2 I_2^0 \tag{S65}$$

where σ_2 is the cross section at the wavelength λ_2. If the condition given in Eq.(S64) is obeyed, the rate constant k_2^0 is given by Eq.(S65) for cases (i2) and (ii). In cases (i1b), (i2) and (ii) we choose I_2^0/I_1^0 and ω/I_1^0 as the two independent control parameters.

Using Eqs.(S7,S10,S17) the control parameters are related to the dimensionless parameters K^0 and θ according to

$$I_1^0 = K^0 \frac{k^\Delta}{\sigma_1} \tag{S66}$$

$$\omega = \theta \left(1 + K^0\right) k^\Delta \tag{S67}$$

for case (i1a). Similarly, for cases (i1b), (i2) and (ii) we obtain

$$\frac{I_2^0}{I_1^0} = \frac{1}{K^0} \frac{\sigma_1}{\sigma_2} \tag{S68}$$

$$\frac{\omega}{I_1^0} = \theta \left(1 + \frac{1}{K^0}\right) \sigma_1 \tag{S69}$$

The resonant values of K^0 and θ at order n are given in Tabs. S1, S2, S3, S4. Resonant control parameter values are obtained in case (i1a) and the other cases by substituting for the resonant values of K^0 and θ into Eqs. (S66,S67) and Eqs. (S68,S69), respectively. The amplitudes of concentration and fluorescence oscillation for the representative photoswitchable species Dronpa-2 in the cases (i1a), (i1b), (i2), and (ii) are given in Figs. S7 and S8 in the control parameter space. The typical behaviors observed in the dimensionless space are recovered in the control parameter space (I_1^0, ω) or $(I_2^0/I_1^0, \omega/I_1^0)$. In particular we numerically proved that the amplitudes at the extremum and the surfaces at half maximum of the resonance peak are unchanged. Hence Figs. S3 and S6 remain relevant in the control parameter spaces. The gap between the resonant parameter values of two reversibly photoswitchable species only depends on the values of their kinetic properties (cross sections σ_1 and σ_2 and thermal rate constant k^Δ) but not on the order n of the harmonics and little on the number of modulated lights.

The Fourier amplitudes with extrema are well-suited observables for discrimination purposes. The parameters that control illumination can be tuned to maximize the contribution of the target and minimize the contributions of interfering species. The nth-order Fourier amplitude of fluorescence oscillation with extrema is further referred to a HIGHLIGHT-n signal. Specifically we harness the resonance properties of A_1 = HIGHLIGHT-1, B_2 = HIGHLIGHT-2, and A_3 = HIGHLIGHT-3. A small width at half maximum is favorable provided that the associated resonant value is large enough to be experimentally detectable. Following Fig. S6 the width of the resonance peak is smaller for cases (i1a) and (i1b). However these cases are associated with the smallest amplitudes and requires a good signal-to-noise ratio. The optimization of both the signal-to-noise ratio and the sharpness of the resonance is obtained for case (ii). Cases (i1b), (i2) and (ii) have advantages with respect to case (i1a). Indeed, the mean light intensity I_1^0 is a fully adjustable parameter that can be chosen large leading to large values of k_1^0. Due to larger values of k_1^0 and k_2^0, cases (i1b), (i2) and (ii) enable faster acquisition than case (i1a), the acquisition time of concentration or fluorescence evolution being of the order of $1/\tau^0$ with τ^0 given in Eq.(S10). Choosing large values of I_1^0 also optimizes the signal-to-noise ratio of fluorescence intensity. Case

(i2) leads to a single resonance at each order and consequently no zero line in the control parameter space. Contrary to case (i2), both cases (i1b) and (ii) show zero lines from the second order.

Figure S9 gives the resonance maps at orders $n = 1, 2, 3$ for cases (i1b) and (ii) and three well-chosen RSFPs (Dronpa, Dronpa-2, and rsFastlime). Interestingly from the experimental point of view, the zero line of one RSFP goes through the resonance of another RSFP and the resonance conditions of orders 1 and 2 nearly coincide in case (i1b). The situation is not as favorable in case (ii) for the chosen RSFPs. We therefore chose case (i1b) to implement the following experimental protocol. For the sake of simplicity we extract the Fourier amplitudes at various orders from the fluorescence evolution recorded for a single control parameter set associated with the resonant condition of the first-order amplitude. Three fluorescence evolutions are recorded for different control parameter values coinciding with the resonance condition of Dronpa, then rsFastLime, and finally Dronpa-2. For each acquisition the first-order quadrature amplitude of fluorescence oscillation and the second-order in-phase amplitude are extracted. At the resonance condition of Dronpa, the detected amplitude contains the contributions of both Dronpa and rsFastLime to the first order whereas the contribution of Dronpa is isolated in the second-order amplitude. Indeed the zero line of the second order amplitude of rsFastLime meets the resonance condition of Dronpa and the resonance condition of Dronpa-2 are sufficiently different for the contribution of Dronpa-2 to be negligible. Similarly, the contribution of Dronpa-2 to the second-order amplitude vanishes in the resonance condition of rsFastLime associated with a point on the zero line of Dronpa-2. The contribution of Dronpa can be neglected. Finally, the contribution of Dronpa-2 is retrieved for its resonance condition, the contributions of Dronpa and rsFastLime being negligible.

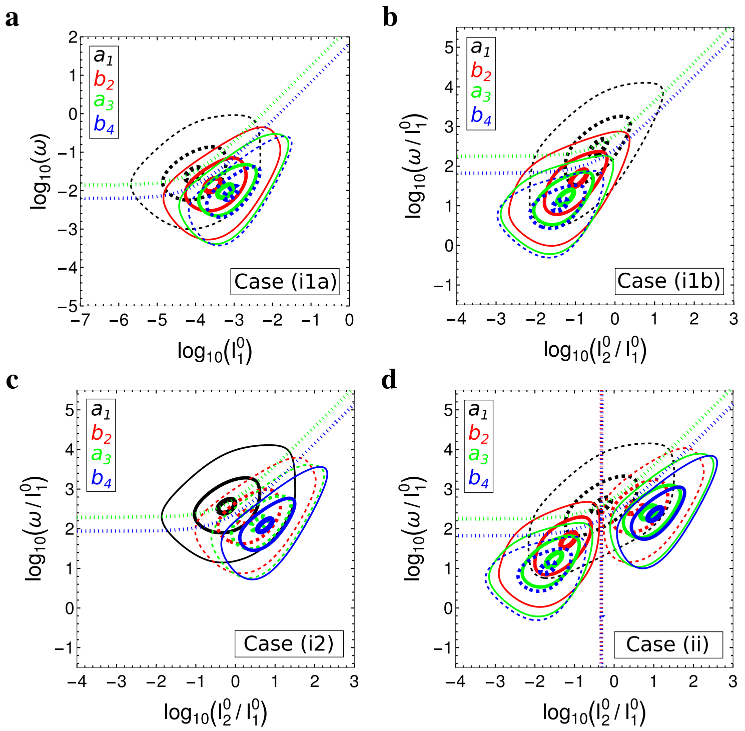

Figure S7: Amplitudes a_1, b_2, a_3, and b_4 of concentration oscillation for the RSFP Dronpa-2 driven by one modulation at the wavelength λ_1 with $I_2^0 = 0$ (a: case (i1a)), with $I_2^0 \neq 0$ (b: case (i1b)), at the wavelength λ_2 with $I_1^0 \neq 0$ (c: case (i2)), and two modulations in antiphase (d: case (ii)). The results are deduced from the numerical solutions of Eqs.(S12-S16) for cases (i1a), (i1b), (i2) and Eqs.(S32-S36) for case (ii) truncated at the fifth order. The zero lines are represented by dotted lines. The other contour lines correspond to 95 % (thick lines), 50 % (medium lines), and 10 % (thin lines) of the extremum value, the positive values being associated with a solid line and the negative values with a dashed line. $\sigma_1 = 196$ m^2.mol^{-1}, $\sigma_2 = 413$ m^2.mol^{-1}, $k^\Delta = 14 \times 10^{-3}$ s^{-1}, and $\alpha = 1$.

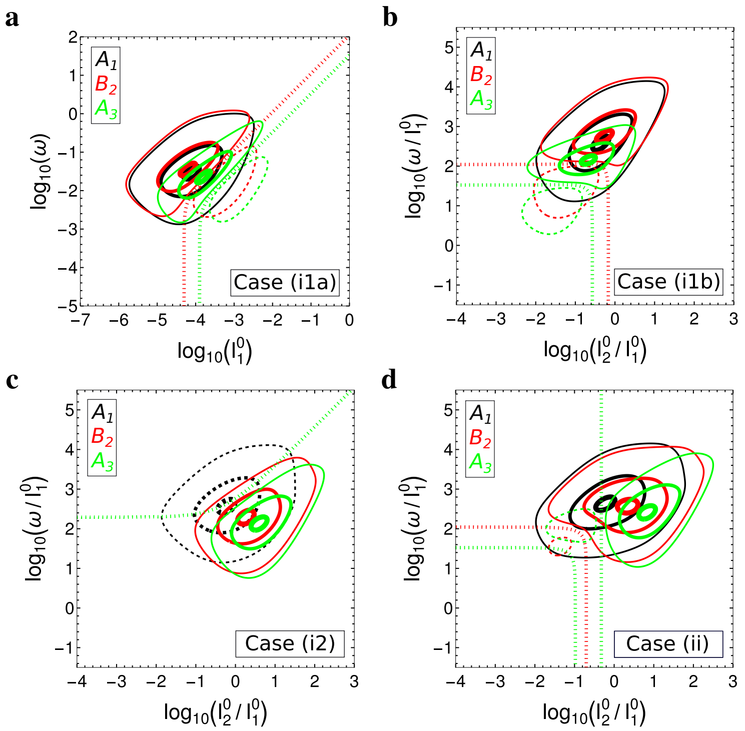

Figure S8: Amplitudes A_1, B_2, and A_3 of fluorescence oscillation for the RSFP Dronpa-2 driven by one modulation at the wavelength λ_1 with $I_2^0 = 0$ (a: case (i1a)), with $I_2^0 \neq 0$ (b: case (i1b)), at the wavelength λ_2 with $I_1^0 \neq 0$ (c: case (i2)), and two modulations in antiphase (d: case (ii)). The results are deduced from Eqs.(S52-S56) in which the amplitudes of concentration oscillation are numerical solutions of Eqs.(S12-S16) for cases (i1a), (i1b), (i2) and Eqs.(S32-S36) for case (ii) truncated at the fifth order. The zero lines are represented by dotted lines. The other contour lines correspond to 95 % (thick lines), 50 % (medium lines), and 10 % (thin lines) of the extremum value, the positive values being associated with a solid line and the negative values with a dashed line. $\sigma_1 = 196$ m^2.mol^{-1}, $\sigma_2 = 413$ m^2.mol^{-1}, $k^\Delta = 14 \times 10^{-3}$ s^{-1}, and $\alpha = 1$.

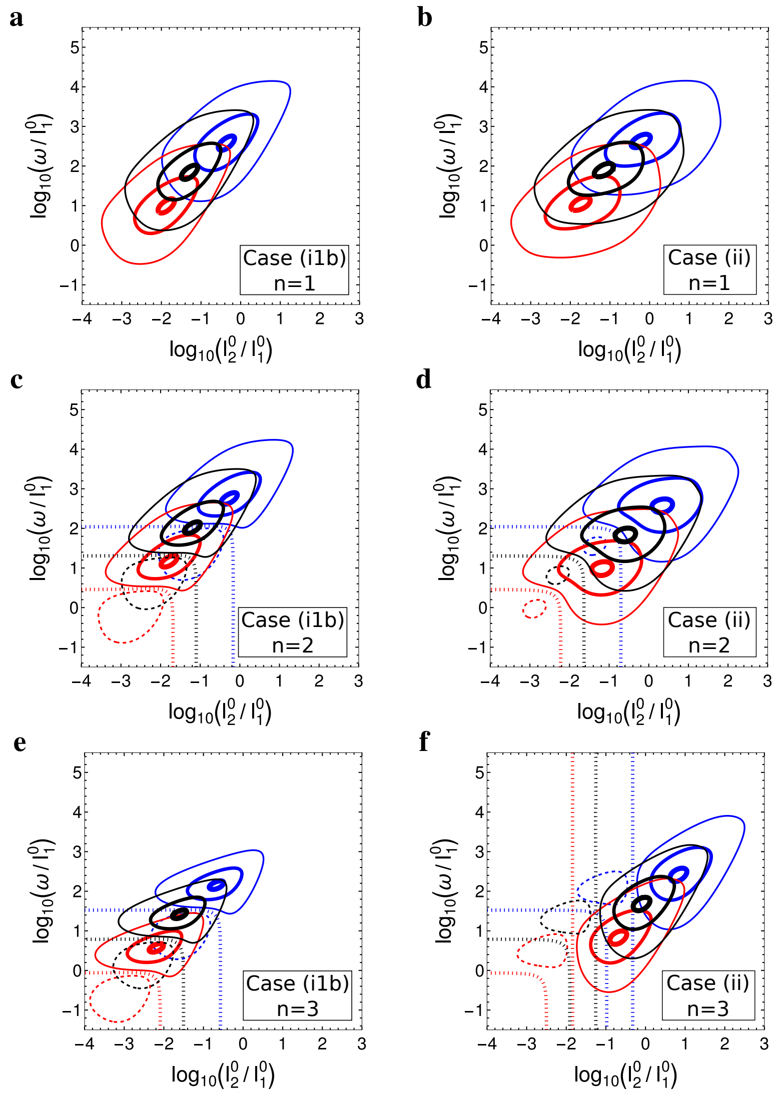

Figure S9: Discrimination of Dronpa-2, rsFastlime, and Dronpa. Amplitudes A_1 (a, b), B_2 (c, d), and A_3 (e, f) of fluorescence oscillation for the RSFPs Dronpa-2 (blue), rsFastLime (black), and Dronpa (red) driven by one modulation at the wavelength λ_1 with $I_2^0 \neq 0$ (a, c, e: case (i1b)) and two modulations in antiphase (b, d, f: case (ii)). The results are deduced from Eqs.(S52-S56) in which the amplitudes of concentration oscillation are numerical solutions of Eqs.(S12-S16) for case (i1b) and Eqs.(S32-S36) for case (ii) truncated at the fifth order. The zero lines are represented by dotted lines. The other contour lines correspond to 95 % (thick lines), 50 % (medium lines), and 10 % (thin lines) of the extremum value, the positive values being associated with a solid line and the negative values with a dashed line. $\sigma_1 = 196$ m^2.mol^{-1}, $\sigma_2 = 413$ m^2.mol^{-1} for Dronpa-2, $\sigma_1 = 36$ m^2.mol^{-1}, $\sigma_2 = 648$ m^2.mol^{-1} for rsFastLime, $\sigma_1 = 5.1$ m^2.mol^{-1}, $\sigma_2 = 354.5$ m^2.mol^{-1} for Dronpa, and $\alpha = 1$.

S2 Experimental Section

S2.1 Protein production and purification

The genes encoding Dronpa-2 and Dronpa-3 were provided by Stefan Jakobs and Atsushi Miyawaki respectively. The genes encoding Dronpa and rsFastLime were provided by Agathe Espagne. The plasmids expressing the fluorescent proteins carrying an N-terminal hexahistidine tag were transformed in E. coli BL21 strain. Cells were grown in Terrific Broth (TB) at 37°C. The expression was induced at 30°C by addition of isopropyl β-D-1-thio-galactopyranoside (IPTG) to a final concentration of 1 mM at OD(600)=0.6. The cells were harvested after 16 h of expression and lysed by sonication in Lysis buffer (50 mM PBS with 150 mM NaCl at pH 7.4, 5 mg/ml DNAse, 5 mM $MgCl_2$ and 1 mM phenylmethylsulfonyl fluoride (PMSF), and a cocktail of protease inhibitors (Sigma Aldrich; S8830)). After lysis, the mixture was incubated on ice for 2 h for DNA digestion. The insoluble material was removed by centrifugation and the supernatant was incubated overnight with Ni-NTA agarose beads (Thermo-fisher) at 4°C in a rotator-mixer. The protein loaded Ni-NTA column was washed twice with 20 column volumes of N1 buffer (50 mM PBS, 300 mM NaCl, 30 mM imidazole, pH 7.4) and twice N2 buffer (50 mM PBS, 150 mM NaCl, 10 mM imidazole, pH 7.4). The bound protein was subsequently eluted with N3 buffer (150 mM PBS pH 7.4, 300 mM imidazole). The protein fractions were eventually dialyzed with cassette Slide-A-Lyzer Dialysis Cassettes (Thermofisher) against 50 mM PBS, 150 mM NaCl pH 7.4.

Production of RSFP-labeled Escherichia coli

Escherichia coli cells from the TOP10 strain were transformed with the RSFP Plasmids by electroporation. The transformed E. coli cells were grown at 37°C in LB broth. When the optical density at 600 nm reached 0.2, expression was induced by addition of isopropyl β-D-1-thio-galactopyranoside (IPTG) to a final concentration of 1 mM. After 4 hours of expression at 30°C, 1 mL aliquots were taken and cells were centrifuged at 8000 rpm for 5 min. After centrifugation, the supernatant was removed and the E. coli cells were washed once with 1 ml of PBS (pH 7.4, 50 mM sodium phosphate, 150 mM NaCl) and then resuspended in 250 μL of PBS buffer.

Preparation of the samples of RSFP-labeled Escherichia coli

Bacteria aliquots (1 mL) from cells culture were centrifuged at 8000 rpm for 5 min. After centrifugation, the supernatant was removed and the E. coli cells were washed once with 1 mL of PBS (pH 7.4, 50 mM sodium phosphate,150 mM NaCl) before they were centrifuged one more time at 8000 rpm for 5 min and resuspended in 500 μL of PBS. 3 μL of the final E. Coli suspension were deposited on a pad of low melting agarose (1% w/w with PBS) deposited on a coverslip. Finally, a cover slide was added on the top of the pad for microscopy observation.

S2.2 Mammalian cell culture and transfection

U2OS cells were incubated at 37°C in a 5% CO_2 in air atmosphere with McCoy's 5A Medium complemented with 10% fetal bovine serum (FBS). Cells were transiently transfected with Genejuice (Merck) according to the manufacturer protocol then washed with Dulbecco's phosphate buffered saline (DPBS) and fixed with 2% paraformaldehyde (PFA) solution.

S2.3 Reagents and solutions

pH 7.4 PBS buffer (50 mM sodium phosphate, 150 mM NaCl) used for all experiments was made up using purified water (Direct-Q 5 apparatus; Millipore, Billerica, MA). By assimilating activity and concentration, the proton concentration of the solutions was directly measured after calibration of the pH meter (Standard pH meter PHM210, Radiometer Analytical equipped with a Radiometer Analytical PHC3359-8 combination pH electrode; Hach, Loveland, CO).

The concentrations of the purified RSFP solutions were determined with a UV-spectrophotometer (Agilent Technologies, Santa Clara, CA). The UV-Vis absorption spectrum of the denatured RSFP solution in 1 M NaOH was recorded from 400 to 600 nm in a 55 μL quartz cuvette with 1.5 mm light path (Hellma Optics, Jena, Germany). The RSFP concentration was extracted from the absorbance at 447 nm by using 44000 $M^{-1}\cdot cm^{-1}$ for the molar absorption coefficient of the deprotonated chromophore.[1] The RSFP solution was subsequently diluted with a PBS buffer (50 mM phosphate, 150 mM NaCl, pH 7.4) to give the RSFP solution at the desired concentration.

S2.4 Microfluidic devices

The microdevices were composed of a circular glass coverslip (0.17 mm thick, 40 mm diameter; Menzel-Glaser, Braunschweig, Germany) and a PDMS stamp (RTV615; General Electrics, Fairfield, CT) including either six (250×125×20 μm^3; in Fig. S18) or four (250 μm × 125 μm × 20 μm; in Fig. 4) chambers separated by 100 μm × 20 μm walls. Each chamber was connected to a sample reservoir punched in the PDMS stamp through a 40 μm × 20 μm channel. Before assembly, the coverslip and the PDMS stamp were rinsed with ethanol and dried under a nitrogen flow. The bottom glass surface of the microdevice was placed on a 0.4 mm thick copper disk in which a 8 mm hole had been opened for further observation with the objective. To fill the micro-chambers with appropriate solutions, the air dissolved in the PDMS was pumped for 3 min at 50 mbar at room temperature and sample solutions were added to each reservoir, which resulted in the autonomous and controlled loading of the device.

S2.5 Epifluorescence setup for multiplexed imaging

We used a home-built epifluorescence microscopy setup. The samples were illuminated using a LXZ1-PB01 LED (Philips Lumileds) filtered at 480±20 nm (F480-40; Semrock, Rochester, NY) and a LHUV-0405 (Philips Lumileds) LED filtered at 405±20 nm (F405-40; Semrock, Rochester, NY) as light sources. Each LED was supplied by a LED driver (LEDD1B, Thorlabs, Newton, NJ). Light levels were sampled with an Arduino compatible card (Teensy 3.5, PJRC). A lens (ACL2520U; Thorlabs, Newton, NJ, $f = 20$ mm) was placed just after each diode to collimate the light sources. The two light beams were next combined thanks to a dichroic mirror (T425LPXR, Chroma, Bellows Falls, VT) and a second pair of lenses was used to focus the light at the back focal plane of the objective after being reflected by the dichroic filter (Di-FF506, Semrock, Rochester, NY). Fluorescence images at 525±15 nm (F525-30; Semrock, Rochester, NY) were acquired for the microdevices with a 10× fluar (NA 0.5, Carl Zeiss AG, Feldbach, Switzerland) objective and for cell imaging with a 50× UPlanApo (NA 0.8, Olympus Corporation, Tokyo, Japan) objective. Objectives were mounted on a home-built microscope equipped with a Luca-R or iXon-897 CCD camera (Andor Technology, Belfast,UK).

S2.6 Epifluorescence setup with inhomogeneous illumination

S2.6.1 Materials

This home-built epi-fluorescence imaging system has been designed to evaluate the enhancement of spatial contrast in non-homogeneous light profiles of HIGHLIGHT by artificially introducing a controllable chromatic aberration between the two excitation wavelengths at 480 and 405 nm (see Fig.S10a). The system integrates two colored LEDs (LXZ1-PB01,

centered at 480 nm and LHUV-0400, centered at 405 nm, Lumileds, NL) as excitation sources, in order to generate a large focal field around the focal plane. Each LED is triggered by a LED driver (DC 4104, Thorlabs, NJ, US). Light from each source is first collimated by lens L1 (AC254-200-A, $f = 200$ mm, Thorlabs, NJ, US) and then reduced by a quasi-afocal system consisting of two lenses (L2: LA1134-A, $f = 60$ mm, L3: LC2679-A, $f = -30$ mm, Thorlabs, Newton, NJ). The reduced beams are combined by a dichroic mirror (T425LPXR, Chroma, Bellows Falls, VT) and then focused by a convergent lens L4 (LA1131-A, $f = 50$ mm, Thorlabs, NJ, US). The focal plane of L4 is conjugated to the focal plane of the objective (UPLSAPO60XW, 60$\times$, NA 1.2, Olympus Corporation, Tokyo, Japan), with the help of the tube lens system L5 and L6 (AC254-200-A, $f = 200$ mm, Thorlabs, NJ, US). The objective along with the tube lenses L5 and L6 give a total magnitude of 100:3, forming an illuminated area of 15 μm at the sample. The position of the imaging plan after L4 can be effectively changed by slightly moving the element L3 back and forth along the optical axis, which makes it possible to defocus each wavelength independently from the focal plane of the objective. According to software simulation, a displacement of the element L3 by 1 mm gives rise to a defocus of 4 μm at the focal plane of the objective. The fluorescence signal from the sample is separated from the excitation lights with another dichroic mirror (T505LPXR, Chroma, Bellows Falls, VT) and then recorded by a CCD camera (Luca-R Andor Technology, Belfast, UK).

S2.6.2 Scheme

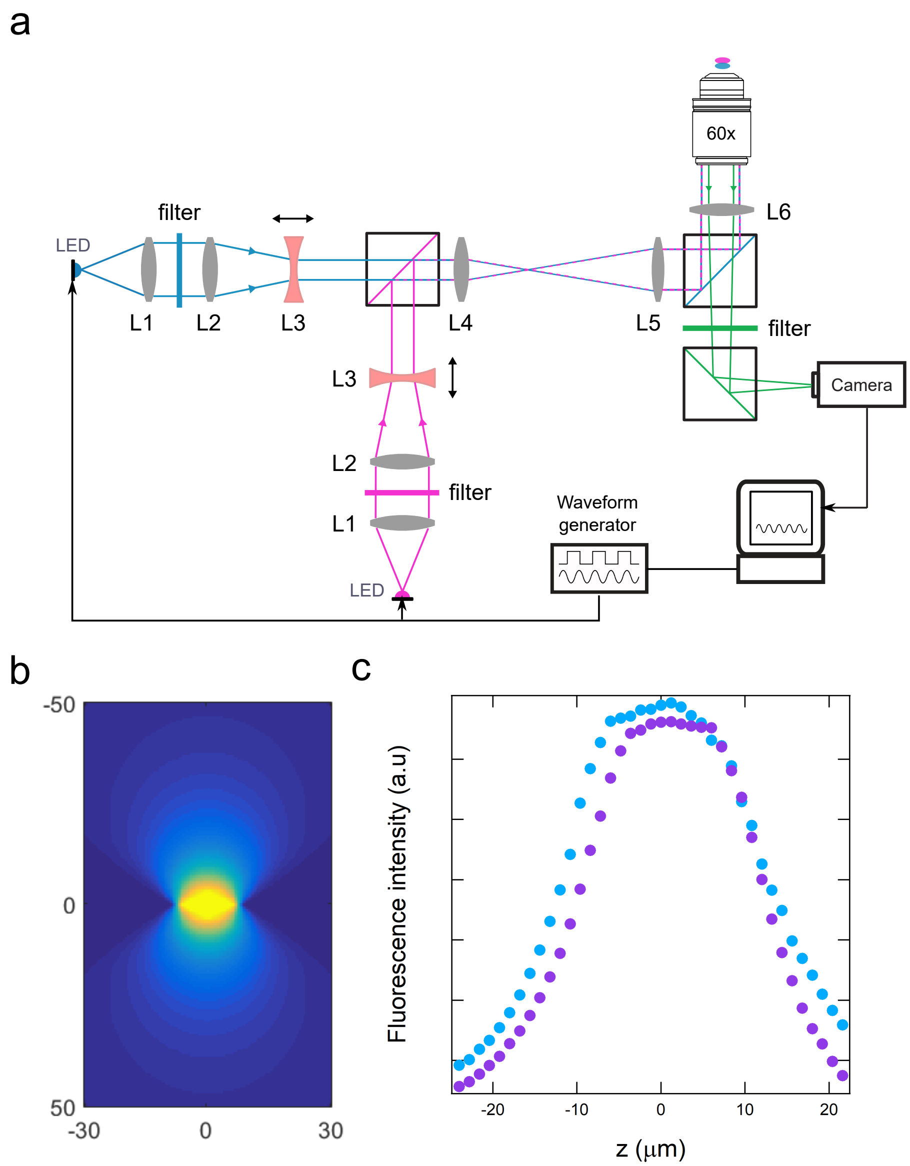

Figure S10: *Fluorescence imaging microscope for enhancing spatial contrast in non-homogeneous light profiles by HIGHLIGHT*. **a**: Optical layout of the microscope and its interface to a PC-based synchronized modulation and imaging acquisition system. This epi-fluorescence imaging system is designed for manual defocusing of the two wavelengths at 480 (in blue) and 405 (in violet) nm; **b**: Simulation of the excitation light intensity in cylindrical coordinates. Essentially similar at 480 and 405 nm, it exhibits strong homogeneous light intensity over a square-based bipyramidal shape with 15 μm diameter and 15 μm height, which acts as a large pixel. Illumination is predicted to be laterally restricted in the focal plane and to axially decay over ten μm from the focal plane; **c**: Dependence of the normalized light intensity at 480 (blue disks) and 405 (violet disks) nm measured along the optical axis.

S2.6.3 Inhomogeneous illumination

Model of light profile The light distribution along the optical axis around the intermediate image on the focal plane in a medium of refractive index n was computed by making several approximations:

- The intensity of the image is considered to be constant across the image plane. This results from the uniformity of

the LED source itself;

- The radiance L of the image is angularly constant within the aperture angle of the objective, because the LED source can be considered as a lambertian source;
- Assuming that the geometric aberration of the system is negligible, the light intensity distribution is symmetrical to the image plane (mirror symmetry, $I(-z) = I(+z)$).

We first analyzed the spatial profile of the light intensity around the focal plane of the objective, where a round shaped image of the LED source with a R_0 radius is projected. Since the light distribution is rotationally symmetric to the optical axis and mirrorly symmetric to the focal plane, we only computed the light irradiance I at medium points (r, z) identified by their cylindrical coordinates with origin set at the center of the focal plane (see Fig. S11).

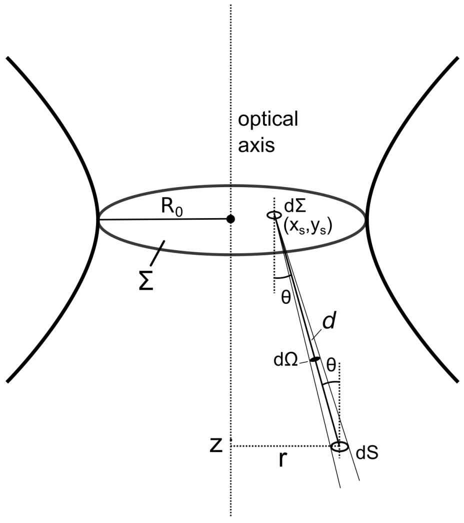

Figure S11: Schematic representation of the simulated excitation light intensity.

One has

$$I(r, z) = \frac{\mathrm{d}\Phi_F(r, z)}{\mathrm{d}S} \tag{S70}$$

where r is the distance to the optical axis of the system, z is the distance to the focal plane, and $\Phi_F(r, z)$ is the photon flux passing through an element of area $\mathrm{d}S$ at (r, z).

When analyzing the points (r, z) close to the image in the focal plane ($z \sim 2R_0$), the intermediate image is considered as a surface source Σ. Each element of surface $\mathrm{d}\Sigma$ at $(x_s, y_s, 0)$ is considered Lambertian within an acceptance angle θ_m. The radiance can be expressed as:

$$L(\theta) = \begin{cases} L_s & \theta \leqslant \theta_m \\ 0 & \theta > \theta_m \end{cases}$$

where θ is the angle between the light ray and the normal of the surface Σ. The acceptance angle is defined by the numerical aperture of the objective in use, NA= $n \sin(\theta_m) = 1.2$, where n is the refractive index of the medium around the fiber.

The flux received by $\mathrm{d}S$ at (r, z) from the source element $\mathrm{d}\Sigma$ at $(x_s, y_s, 0)$ is:

$$\mathrm{d}^2\Phi_F = L(\theta)10^{-\frac{d}{d_c}}\mathrm{d}^2G = L(\theta)10^{-\frac{d}{d_c}}\frac{\mathrm{d}S\mathrm{d}\Sigma\cos^2\theta}{d^2} = L(\theta)10^{-\frac{d}{d_c}}\frac{\mathrm{d}S\mathrm{d}\Sigma\cos^4\theta}{z^2} \tag{S71}$$

where d_c is a typical length. In Eq.(S71), G is the *etendue* defined as

$$\mathrm{d}^2 G = \mathrm{d}\Sigma \cos\theta \mathrm{d}\Omega_\Sigma = \frac{\mathrm{d}\Sigma \mathrm{d}S \cos^2\theta}{d^2} \tag{S72}$$

where d is the distance between $\mathrm{d}\Sigma$ and $\mathrm{d}S$, and $\cos\theta = \frac{z}{d} = \frac{z}{\sqrt{(r-x_s)^2+y_s^2+z^2}}$ (see Fig. S11).

Finally we get the irradiance at (r, z) from the entire surface Σ:

$$\begin{aligned} I(r,z) &= \frac{\mathrm{d}\Phi_F(r,z)}{\mathrm{d}S} = \frac{1}{\mathrm{d}S}\int_\Sigma \mathrm{d}^2\Phi_F(r,z) \\ &= \frac{1}{\mathrm{d}S}\int_\Sigma \frac{L(\theta)\mathrm{d}\Sigma\mathrm{d}S\cos^2\theta}{d^2}10^{-\frac{d}{d_c}} = \int_\Sigma \frac{L(\theta)\cos^4\theta}{z^2}10^{-\frac{d}{d_c}}\mathrm{d}\Sigma \end{aligned} \tag{S73}$$

Calculation of the Pre-HIGHLIGHT and HIGHLIGHT-n signals for the model of inhomogeneous light profile
We neglect possible chromatic aberration of the fluorescence microscope and assume that the medium exhibits similar absorption and scattering of light at the two wavelengths. Consequently illuminations have the same spatial dependence at both wavelengths and the ratio $I_2^0(r,z)/I_1^0(r,z)$ is constant. In the following, we consider that the HIGHLIGHT-1 resonance condition is fulfilled in the focal plane on the optical axis ($r = z = 0$). According to Eqs. (S68,S69) for $K^0 = \theta = 1$ we have

$$\frac{I_2^0(r,z)}{I_1^0(r,z)} = \frac{\sigma_1}{\sigma_2} \tag{S74}$$

$$\frac{\omega}{I_1^0(0,0)} = 2\sigma_1 \tag{S75}$$

The condition given in Eq. (S75) is not obeyed apart from the intersection between the optical axis and the focal plane. The HIGHLIGHT-n signals display a better axial selectivity than the Pre-HIGHLIGHT signal.

The spatial dependence of the Pre-HIGHLIGHT and HIGHLIGHT-n signals are computed using Eqs. (S19,S20,S23–S29) and Eqs. (S52–S56). The dependence of the parameters K^0 and θ is deduced from Eqs. (S68,S69) and the light profile is given in Eq. (S73) with an experimentally evaluated typical length d_c. The results are given in Fig. S12. The areas in which the HIGHLIGHT-n signals are significant are smaller than in the Pre-HIGHLIGHT signal. With the present setup which projects the contributions of all fluorophores located along the optical axis, the HIGHLIGHT-n signals are dominated by the contributions of the fluorophores located close to the focal plane as shown in Figure 5.

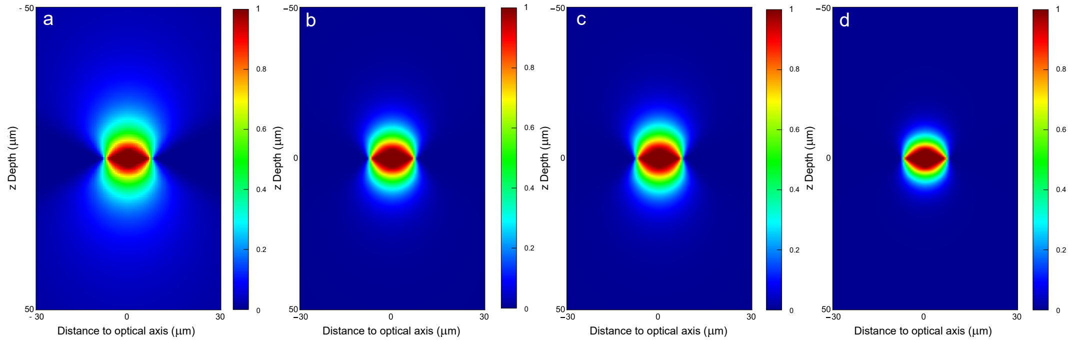

Figure S12: Spatial dependence of the scaled Pre-HIGHLIGHT (**a**), HIGHLIGHT-1 (**b**), HIGHLIGHT-2 (**c**) and HIGHLIGHT-3 (**d**) signals after introducing the light profiles in the analytical expressions of the HIGHLIGHT signals with sine-wave light modulation at $\lambda_1 = 480$ nm and constant light at $\lambda_2 = 405$ nm.

S2.6.4 Characterization of the illumination profile

In order to study the excitation profiles from the LEDs at λ_1 = 488 nm and λ_2 = 405 nm, we imaged a 2 μm-thick layer of 5 μM fluorescein solution in pH 7.4 PBS buffer (sandwiched between two 170 μm-thick glass plates) at different axial positions ranging from 0 to 21.6 μm. Fig. S10c has been obtained after measuring the signal emission intensity for each axial position.

S2.6.5 Samples for confocality validation

Dronpa-2-containing samples of defined thickness have been obtained by sandwiching Dronpa-2 solutions between two 170 μm-thick glass slides, which have been treated with air plasma cleaner to increase their surface hydrophilicity. In order to control the sample thicknesses below the 20 μm range, we used either polystyrene beads (carboxylate microsphere, Polybead, Polysciences Inc, Us) or magnetic polystyrene beads (Magnetic polystyrene particles micromer, Micromer-M, Micromod, Germany) with 4, 6, 12 and 20 μm diameters as spacers. The commercial suspension of beads was first diluted in PBS to the thousandth. Then the resulting suspension was uniformly suspended by sonicating. It was eventually mixed with the Dronpa-2 solution for sandwiching. In order to get the 50, 80, and 120 μm sample thicknesses, we exploited a double sided tape as home-made imaging spacer and a 120 μm-thick commercially available spacer (SecureSeal, Sigma-Aldrich, Grace Bio-Lab, USA).

S2.7 Generation of pure sine-wave light modulation

Light emitted from a LED was modulated sinusoidally and filtered with a bandpass filter (ZET 405-20x, Chroma Technology, Bellows Falls, VT, for the violet LED or ET 480-20x, Chroma Technology for the blue LED). An optical fiber (M38L01, Thorlabs, Newton, NJ) was used to collect part of the emitted light that was next focused with a $f = 40$ mm lens onto the sensor of a silicon photomultiplier module (C13366-3050GA, Hamamatsu Photonics K.K., Hamamatsu City, Japan). Light levels were sampled with an Arduino compatible card (Teensy 3.5, PJRC) which calculated the Fourier Transform of the silicon photomultiplier signal to retrieve the harmonic content of the modulated light. The first harmonics up to the 10th order were introduced with a 180° phase shift in the voltage modulation delivered by a DAC output of the Teensy 3.5 which controlled the electric current delivered by a LED driver (LEDD1B, Thorlabs) to the LED chip. After a few iterations (typically 10), the harmonic content of modulated light emitted from the LED chip was efficiently damped: the amplitude of each harmonic was measured independently by our EM-CCD camera (Luca-R, Andor Technology, Belfast, Northern Ireland) and was found to be less than 0.75% of the amplitude term at fundamental frequency (see Fig. S13 and S14).

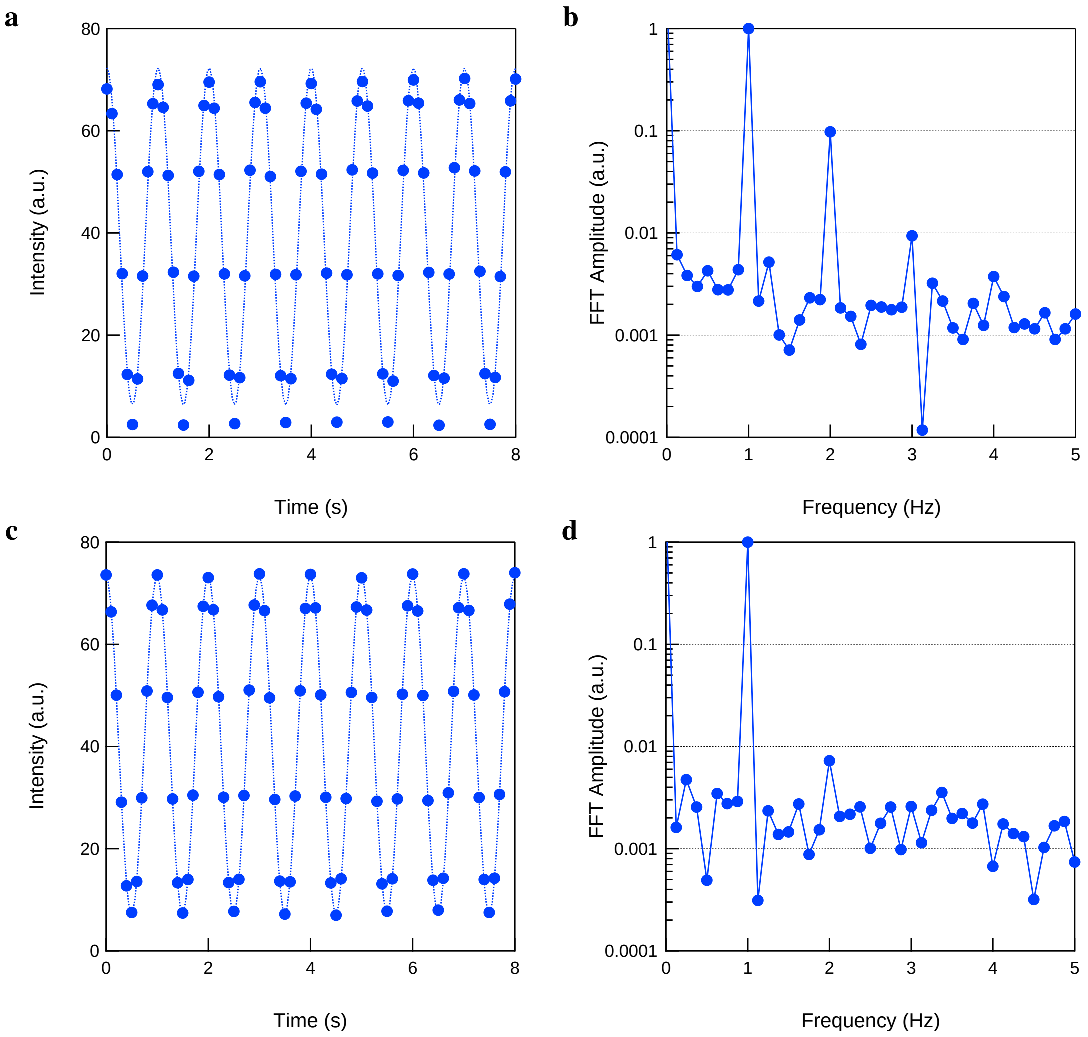

Figure S13: Generation of pure sine-wave light from the LED at 480 nm. **a**,**c**: Evolution of the light intensity of the modulated LED light before (**a**) and after (**c**) harmonic correction of the sine-wave tension from the generator supplying the LED; **b**,**d**: Fourier transforms of the signals displayed in **a**,**c**.

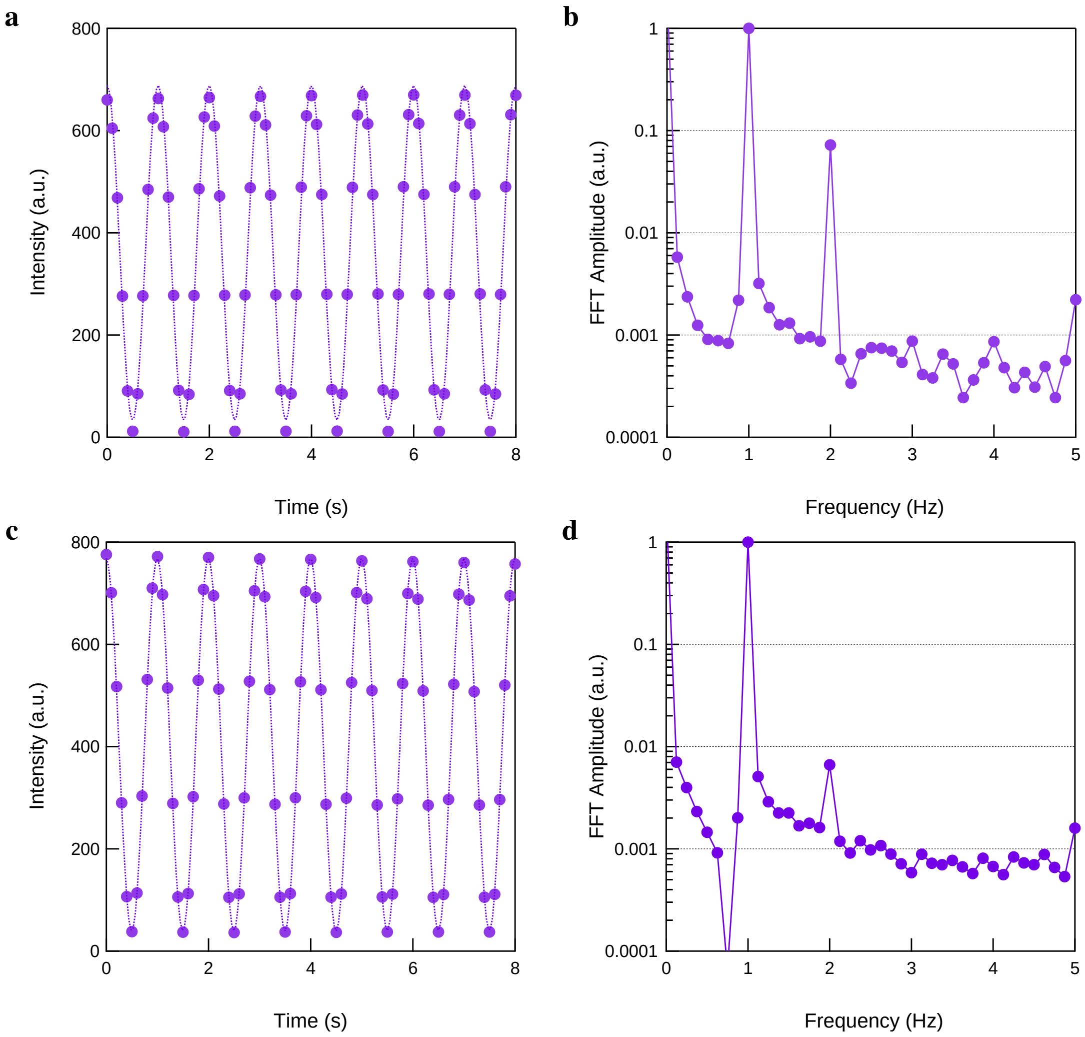

Figure S14: Generation of pure sine-wave light from the LED at 405 nm. **a**,**c**: Evolution of the light intensity of the modulated LED light before (**a**) and after (**c**) harmonic correction of the sine-wave tension from the generator supplying the LED; **b**,**d**: Fourier transforms of the signals displayed in **a**,**c**.

S2.8 Video acquisition

In the imaging experiments, we record films for m periods of light modulation (m is an integer). The acquisition frequency of the camera is set to obtain $2N$ (N is an integer) frames per period of modulation. Thus the acquisition frequency is $f_s = 2Nf_m$, where f_m is the modulation frequency of the excitation light. The fluorescence emission acquired at pixel (*x*,*y*) of the kth frame is equal to

$$I_{\mathrm{F}}(x,y,k) = T_s\Bigg(I_F^0(x,y) + \sum_{n=1}^{N}\Bigg\{B_n(x,y)\sin\left[\frac{\pi n f_s}{N}\left(\frac{k}{f_s}+\phi_{\mathrm{acq}}\right)\right] + A_n(x,y)\cos\left[\frac{\pi n f_s}{N}\left(\frac{k}{f_s}+\phi_{\mathrm{acq}}\right)\right]\Bigg\}\Bigg) \tag{S76}$$

where $T_s = \frac{1}{f_s}$ is the exposure time of one frame, $B_n(x,y)$ and $A_n(x,y)$ are the n-th in-phase and quadrature Fourier amplitudes of the fluorescence signal oscillating around the average value $I_F^0(x,y)$. The phase ϕ_{acq} may originate from distinct starting times for the light modulation and the acquisition of the camera and can be easily calibrated using the

fluorescence emission from instantaneously responding fluorophores (such as EGFP or Fluorescein).

Pre-processing is performed to compensate for possible photobleaching of fluorophores. Assuming that photobleaching linearly decays, we evaluate the compensation factor $K(x,y)$ from the average of two successive periods

$$K(x,y) = \frac{\langle I_F(x,y,k)\rangle_{k=2N}^{4N-1} - \langle I_F(x,y,k)\rangle_{k=0}^{2N-1}}{2N} \tag{S77}$$

Then the frames of the same two periods are corrected according to

$$I_F^{\text{corr}}(x,y,k) = I_F(x,y,k) - K(x,y) \times k \tag{S78}$$

The procedure is repeated for the whole video.

The Pre-HIGHLIGHT image $I_F^0(x,y)$ is calculated by averaging the frames over the whole film

$$I_F^0(x,y) = \frac{f_s}{2mN} \sum_{k=0}^{2\text{mN}-1} I_F^{\text{corr}}(x,y,k) \tag{S79}$$

The HIGHLIGHT-n signals are retrieved according to

$$A_1 = \frac{f_s}{mN} \sum_{k=0}^{2\text{mN}-1} \left\{ I_F^{\text{corr}}(x,y,k) \times \cos\left[\frac{\pi f_s}{N}\left(\frac{k}{f_s} + \phi_{\text{acq}}\right)\right]\right\} \tag{S80}$$

$$B_2 = \frac{f_s}{mN} \sum_{k=0}^{2\text{mN}-1} \left\{ I_F^{\text{corr}}(x,y,k) \times \sin\left[2\frac{\pi f_s}{N}\left(\frac{k}{f_s} + \phi_{\text{acq}}\right)\right]\right\} \tag{S81}$$

$$A_3 = \frac{f_s}{mN} \sum_{k=0}^{2\text{mN}-1} \left\{ I_F^{\text{corr}}(x,y,k) \times \cos\left[3\frac{\pi f_s}{N}\left(\frac{k}{f_s} + \phi_{\text{acq}}\right)\right]\right\}. \tag{S82}$$

In section S2.10, we provide the Matlab code, which we used to compute the HIGHLIGHT-n signals in our imaging experiments.

S2.9 Calibration of light intensities

HIGHLIGHT implementation requires knowing the intensity of the two excitation lights at 480 and 405 nm in the sample in order to set the control parameters at the resonance condition for the targeted RSFP. Instead of using a powermeter, we relied on the photoswitching kinetics of a photochemically well-characterized RSFP (Dronpa-2 at pH 7.4) for measuring light intensities at which the kinetics is well-accounted by a two-state model (see section S1.1). Such an actinometric measurement is photochemically reliable up to 10 ein.m^{-2}.s^{-1} light intensities at the wavelengths $\lambda_1 = 480$ nm and $\lambda_2 = 405$ nm respectively. The calibration of the light intensities exploits light jumps on Dronpa-2 samples (calibrating sample: 6 μm-thick layer of 20 μM Dronpa-2 solution in pH 7.4 PBS buffer sandwiched between two 130 μm-thick glass slides). We use the reported values of $\sigma_1 = 196$ m^{2}mol^{-1}, $\sigma_2 = 413$ m^{2}mol^{-1}, and $k^\Delta = 0.014\ s^{-1}$ for Dronpa-2.[3]

- As a preliminary step of the calibration protocol, the Dronpa-2 solution is illuminated with the 405 nm LED ($I_2^0 = 0.01$ ein.m^{-2}.s^{-1} for 2 min) to secure that Dronpa-2 is initially in its thermodynamically stable state C_1.

- In a first step, the sample is submitted to constant light intensity I_1^0 at $\lambda_1 = 480$ nm. Dronpa-2 switches from the bright state C_1 to the dark state C_2 (see Fig. S15a). The evolution of fluorescence is averaged over space and compared to

$$I_{\text{F}}(t) = A + B\exp\left(-\frac{t}{\tau}\right) \tag{S83}$$

where A, B, and τ are used as fitting parameters. Using Eqs. (S10,S62,S63) relating the characteristic time and the rate constants, we obtain the light intensity at the wavelength λ_1

$$I_1^0 = \frac{1}{\sigma_1}\left(\frac{1}{\tau} - k^\Delta\right) \tag{S84}$$

- In a second step, while maintaining constant the illumination I_1^0 at the wavelength $\lambda_1 = 480$ nm, we submit the sample to to a constant light I_2^0 at the wavelength $\lambda_2 = 405$ nm in order to drive the C_2 to C_1 reaction and recover fluorescence (see Fig. S15b). The fluorescence evolution is averaged space and compared to the fitting function given in Eq. (S83) leading to an evaluation of the characteristic time τ. Using Eqs. (S10,S62,S63,S65) relating the characteristic time and the rate constants, we obtain the light intensity at the wavelength λ_2

$$I_2^0 = \frac{1}{\sigma_2}\left(\frac{1}{\tau} - k^\Delta - \sigma_1 I_1^0\right) \tag{S85}$$

where the light intensity I_1 has been evaluated in the first step.

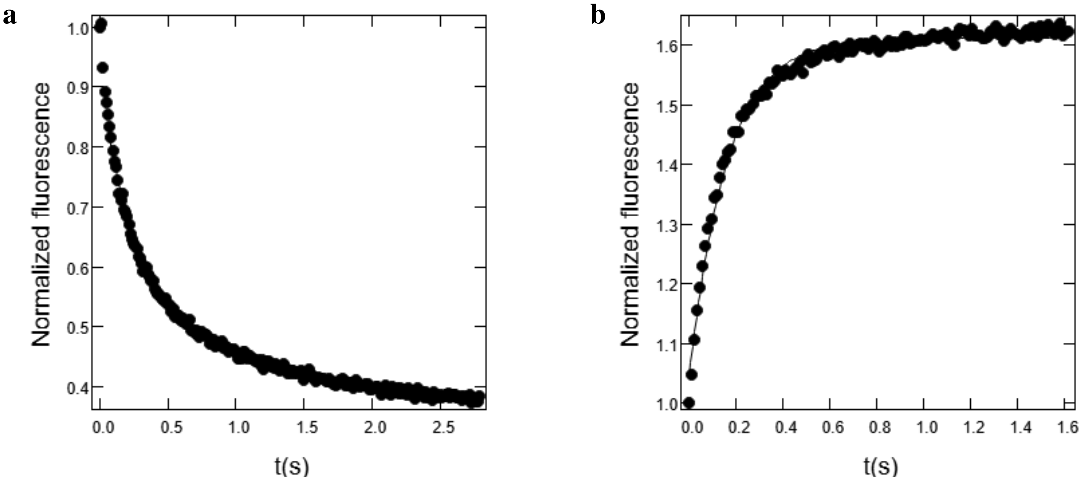

Figure S15: Calibration of the light intensities using the photoisomerization kinetics of Dronpa-2. **a**: Fluorescence evolution of a 20 μM Dronpa-2 solution in pH 7.4 PBS buffer submitted to illumination at $\lambda_1 = 480$ nm ($I_1^0 = 4.8 \times 10^{-2}$ Ein.m^{-2}s^{-1}); **b**: Fluorescence evolution of a 20 μM Dronpa-2 solution in pH 7.4 PBS buffer submitted to illumination at both $\lambda_1 = 480$ and $\lambda_2 = 405$ nm ($I_1^0 = 1.6 \times 10^{-2}$ Ein.m^{-2}s^{-1} and I_2^0 =7.6$\times$10^{-3} Ein.m^{-2}s^{-1}). Disks: Experimental data; solid lines: Fitting function given in Eq. (S83). $T = 298$ K.

S2.10 Matlab code for extracting the HIGHLIGHT signals

```
function [IF0,IF1in,IF1out,IF2in,IF2out,Phase]=highlight(filename,period,phi_acq,n_per,skip_per)
% Computes pre-HIGHLIGHT (IF0) and HIGHLIGHT responses IF1out and IF2in
% We assume movie acquired is already loaded in matlab as 'filename'
% (as camera manufacturers usually provide code to import files)
% 'period' is the period of the light excitation at fundamental frequency in frame number
% 'phi_acq' is the phase delay between the dates of camera recording
% and light excitation previously calibrated at first order with fluorescein or EGFP
% 'n_per' is the total number of periods used for calculation,
% an even number of periods is considered (typically 8)
% 'skip_per' is the number of periods to skip before calculation
```

```
npts = period*n_per;
first = skip_per*period;
[X,Y,Z] = size(filename);
Holder = zeros(X,Y,npts);
IF1in = zeros(X,Y);
IF1out = zeros(X,Y);
IF2in = zeros(X,Y);
IF2out = zeros(X,Y);

for i=1:npts
     Holder(:,:,i)=filename(:,:,i+first);
end

%%%%%%%%%%%%%%%%%%%%%%%%%%%%%%%%%%%%%%%%%%%%%%%%%%%%%%%%%%
%%  Photobleaching correction assuming a linear decay
%%%%%%%%%%%%%%%%%%%%%%%%%%%%%%%%%%%%%%%%%%%%%%%%%%%%%%%%%%

% Calculate the average signal over the first half of 2n periods
 linePoint1=mean(Holder(:,:,1:((npts)/2)),3);
%Calculate the average signal over the last half of 2n periods
 linePoint2=mean(Holder(:,:,((npts)/2+1):end),3);
% Calculate the slope B(x,y)
 slope=(linePoint2-linePoint1)/(npts/2);
%Correction for photobleaching
  for i=1:npts
     Holder(:,:,i)=(Holder(:,:,i)-slope.*i);
  end

%%%%%%%%%%%%%%%%%%%%%%%%%%%%%%%%%%%%%%%%%%%%%%%%%%%%%%%%%%
%%  Data processing
%%%%%%%%%%%%%%%%%%%%%%%%%%%%%%%%%%%%%%%%%%%%%%%%%%%%%%%%%%

% Compute pre-HIGHLIGHT image
IF0=mean(Holder,3); % pre-HIGHLIGHT image

% Compute HIGHLIGHT images
for i=1:npts
    IF1in(:,:)= (IF1in(:,:) + ((Holder(:,:,i)).*sin(2*pi*(i-1)/period + phi_acq)));
    IF1out(:,:)= (IF1out(:,:) + ((Holder(:,:,i)).*cos(2*pi*(i-1)/period + phi_acq)));
    IF2in(:,:) = (IF2in(:,:) + (Holder(:,:,i)).*sin(4*pi*(i-1)/period + 2*phi_acq));
    IF2out(:,:) = (IF2out(:,:) + (Holder(:,:,i)).*cos(4*pi*(i-1)/period + 2*phi_acq));
end

IF1in = 2.*IF1in./npts; % HIGHLIGHT 1in image
IF1out = 2.*IF1out./npts; % HIGHLIGHT 1out image
IF2in = 2.*IF2in./npts; % HIGHLIGHT 2in image
IF2out = 2.*IF2out./npts; % HIGHLIGHT 2out image
Phase = atan(IF1out./IF1in); %to calibrate phase with Fluorescein or EGFP


%%%%%%%%%%%%%%%%%%%%%%%%%%%%%%%%%%%%%%%%%%%%%%%%%%%%%%%%%%
%%  Display Pre-HIGHLIGHT
%%%%%%%%%%%%%%%%%%%%%%%%%%%%%%%%%%%%%%%%%%%%%%%%%%%%%%%%%%

CameraDark = 508.12; %Black level of the camera, Change to match your model
IF0=IF0-CameraDark; %We substract the black level
```

```
figure;imagesc(IF0);colormap(gray(4096));colorbar;axis equal tight;

%%%%%%%%%%%%%%%%%%%%%%%%%%%%%%%%%%%%%%%%%%%%%%%%%%%%%%%%%%%
%%  Display HIGHLIGHT
%%%%%%%%%%%%%%%%%%%%%%%%%%%%%%%%%%%%%%%%%%%%%%%%%%%%%%%%%%%

%Generate 1in image
figure;imagesc(IF1in);colormap(gray(4096));colorbar;
%Generate 1out image
figure;imagesc(IF1out);colormap(gray(4096));colorbar;
%Generate 2in image
figure;imagesc(IF2in);colormap(gray(4096));colorbar;
%Generate 2out image
figure;imagesc(IF2out);colormap(gray(4096));colorbar;
end
```

S2.11 Cell imaging

This series of experiments has been performed using sine-wave modulation of large amplitude (100%) at $\lambda_1 = 480$ nm and constant light at $\lambda_2 = 405$ nm. The fluorescence intensity $I_\mathrm{F}(x, y, t)$ of each pixel was analyzed with Eqs.(S79–S82) to provide the $I_F^0(x, y)$ (pre-HIGHLIGHT) and the $A_1(x, y)$ (HIGHLIGHT-1), $B_2(x, y)$ (HIGHLIGHT-2), $A_3(x, y)$ (HIGHLIGHT-3) images. To reduce noise, the images have been spatially averaged over a 3-pixel square. The acquisition parameters used for HIGHLIGHT imaging of cells are reported in Tab. S6.

S2.12 Confocal experiments

The images displayed in Fig. 5a–f and Fig. S23a–f devoted to evidence the enhancement of spatial contrast in non-homogeneous light profiles by HIGHLIGHT were recorded for a 10-μM Dronpa-2 solution in pH 7.4 PBS buffer for different sample thicknesses z using sine-wave modulation of large amplitude (100%) at $\lambda_1 = 480$ nm ($\lambda_2 = 405$ nm, resp.) and constant light at $\lambda_2 = 405$ nm ($\lambda_1 = 480$ nm, resp.). The dependence of the signal intensities on z shown in Fig. 5g and Fig. S23g have been obtained after spatial averaging of the images over a region of interest of 400 pixels (equivalent to a square of 6 μm in the sample).

S2.13 Photochemical parameters of the RSFPs

Table S5: Photochemical parameters of Dronpa, Dronpa-2, Dronpa-3, and rsFastLime at pH 7.5 and at 37 °C. σ_1 and σ_2 designate the photoswitching cross sections from the bright state C_1 (dark state C_2, resp.) to the dark state C_2 (bright state C_1, resp.) at $\lambda_1 = 480$ nm ($\lambda_2 = 405$ nm, resp.).[3]

RSFP	σ_1 ($m^2.mol^{-1}$)	σ_1 ($m^2.mol^{-1}$)
Dronpa	5,1	354,5
Dronpa-2	196	413
Dronpa-3	29	331
rsFastLime	36	648

S2.14 Acquisition parameters used for the HIGHLIGHT experiments

The acquisition parameters used in the HIGHLIGHT experiments are reported in Tab. S6.

Table S6: Acquisition parameters used to image RSFPs at 25°C in Fig. 2, 3, 4, 5 of the Main Text and Fig. S16, S17, S19, S21, and S23 of the Supporting Information. f_s and f_m refer to the sampling rate and the modulation frequency of the excitation lights. $\alpha = 1$ for all experiments.

Figure	Periods	f_s (Hz)	f_m (Hz)	ω (rad.s^{-1})	I_1^0 (Ein.m^{-2}.s^{-1})	I_2^0 (Ein.m^{-2}.s^{-1})
2e–h	10	12	1	6.3	1.6×10^{-2}	7.6×10^{-2}
2e–h,3a–d,i–k,4c,g,k,5;S18;S19,S21d–f,j–l, S22a–c,g–i,S23	10	12	1	6.3	1.6×10^{-2}	7.6×10^{-3}
2e–h	10	12	1	6.3	1.6×10^{-2}	7.6×10^{-4}
2e–h	10	12	1	6.3	1.6×10^{-2}	7.6×10^{-5}
2e–h	10	1.20	0.1	0.6	1.6×10^{-2}	7.6×10^{-3}
2e–h	10	12	1	6.3	1.6×10^{-2}	7.6×10^{-3}
2e–h	10	120	10	63	1.6×10^{-2}	7.6×10^{-3}
3m–o,4b,f,j; S22d–f,j–l	40	0.3	0.025	0.16	1.5×10^{-2}	2.2×10^{-4}
3e–h, 4d,h,l;S17,S21a–c,g–i	10	12	1	6.3	8.7×10^{-2}	4.8×10^{-3}
S16	10	12	1	6.3	0.1	9.4×10^{-3}

S2.15 Softwares

Data treatment, image analysis and theoretical computations were performed using Igor Pro (WaveMetrics), MATLAB (The MathWorks), Gnuplot and Mathematica (Wolfram Research) softwares.

S3 Supplementary Figures

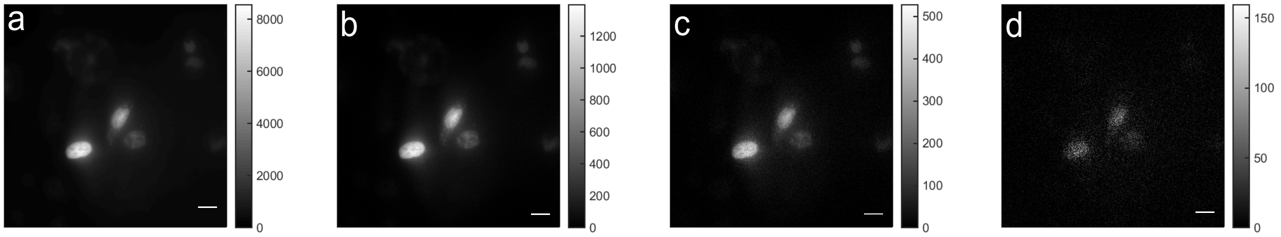

Figure S16: HIGHLIGHT-n discrimination of Dronpa-3. Pre-HIGHLIGHT (**a**), HIGHLIGHT-1 (**b**), HIGHLIGHT-2 (**c**), HIGHLIGHT-3 (**d**) images of Dronpa-3. System: Fixed U2OS cells expressing H2B-Dronpa-3 (at the nucleus). The images were recorded at 298 K. Scale bars: 10 μm. The control parameters are set at the resonance of HIGHLIGHT-1 for Dronpa-3. See Tab. S6 in Supporting Information for the acquisition conditions.

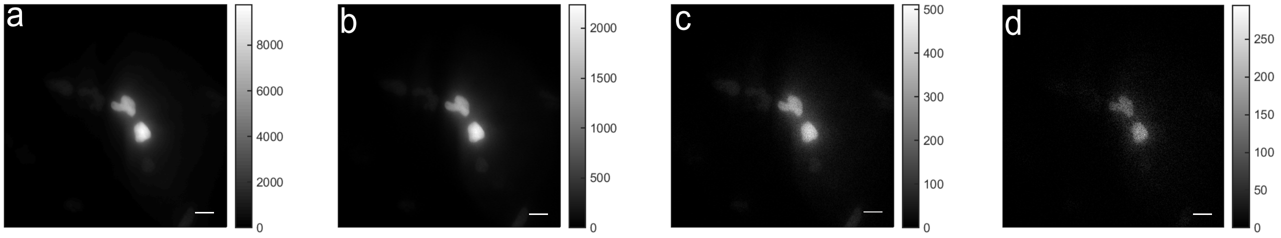

Figure S17: HIGHLIGHT-n discrimination of rsFastLime. Pre-HIGHLIGHT (**a**), HIGHLIGHT-1 (**b**), HIGHLIGHT-2 (**c**), HIGHLIGHT-3 (**d**) image of rsFastLime. System: Fixed U2OS cells expressing H2B-rsFastLime (at the nucleus). The images were recorded at 298 K. Scale bars: 10 μm. The control parameters are set at the resonance of HIGHLIGHT-1 for rsFastLime. See Tab. S6 in Supporting Information for the acquisition conditions.

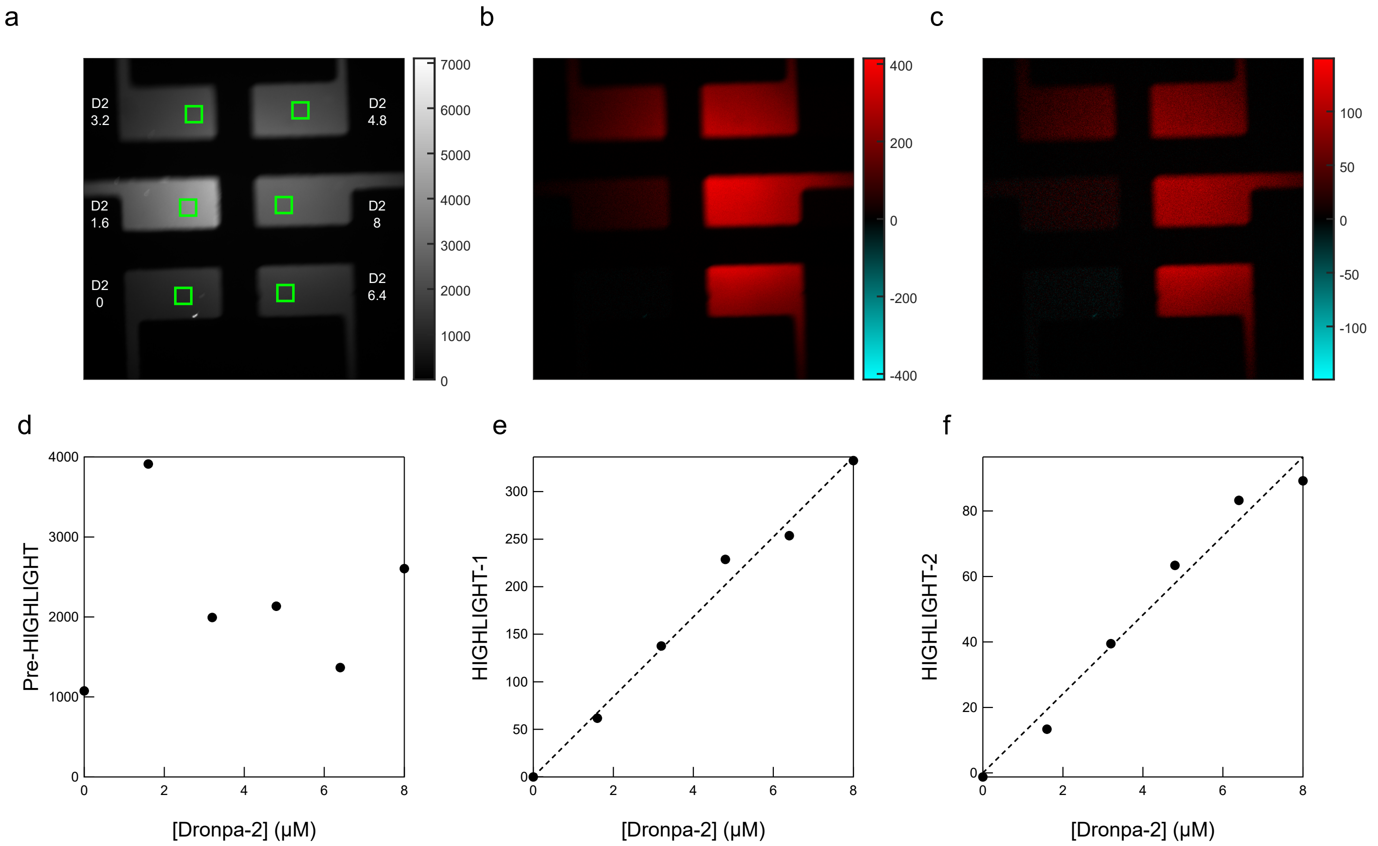

Figure S18: Quantitative imaging of Dronpa-2 in a microfluidic device. A microfluidic device with six rectangular chambers ($250 \times 125 \times 20$ μm^3) was filled with mixtures of Dronpa-2 (D2) and EGFP introduced in various concentrations (the numbers in **a** indicate the concentration of Dronpa-2 in μM in each microchamber). For each mixture, the concentration of EFGP was adjusted to get similar fluorescence intensities from the microchambers in the Pre-HIGHLIGHT image. The pre-HIGHLIGHT (**a**), HIGHLIGHT-1 (**b**), and HIGHLIGHT-2 (**c**) images of the microfluidic device were recorded with a 10 $\times$ objective at λ_{em} = 525 nm under sine-wave light at $\lambda_1 = 480$ nm and constant light at $\lambda_2 = 405$ nm. Dependence of the average fluorescence intensity – pre-HIGHLIGHT (**d**), and average intensity of the HIGHLIGHT-1 (**e**) and HIGHLIGHT-2 (**f**) responses as a function of the Dronpa-2 concentration. The analyzed regions of interest in **d**–**f** are delimited by the green rectangles in **a**. The images were recorded at 298 K. The control parameters are set at the resonance of HIGHLIGHT-1 for Dronpa-2. See Tab. S6 in Supporting Information for the acquisition conditions.

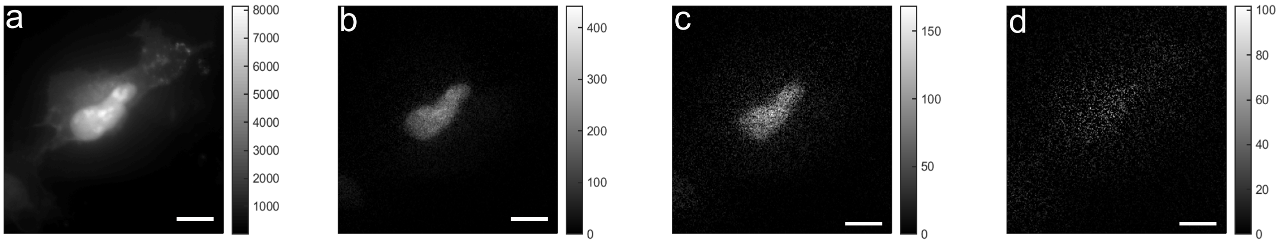

Figure S19: *HIGHLIGHT-n eliminate spectral interferences in fixed cells*. Pre-HIGHLIGHT (**a**), HIGHLIGHT-1 (**b**), HIGHLIGHT-2 (**c**), HIGHLIGHT-3 (**d**) images of Dronpa-2 and EGFP. System: Fixed U2OS cells expressing H2B-Dronpa-2 (at the nucleus) and Lyn11-EGFP (at the cell membrane). The images were recorded at 298 K. Scale bars: 10 μm. The control parameters are set at the resonance of HIGHLIGHT-1 for Dronpa-2. See Tab. S6 in Supporting Information for the acquisition conditions.

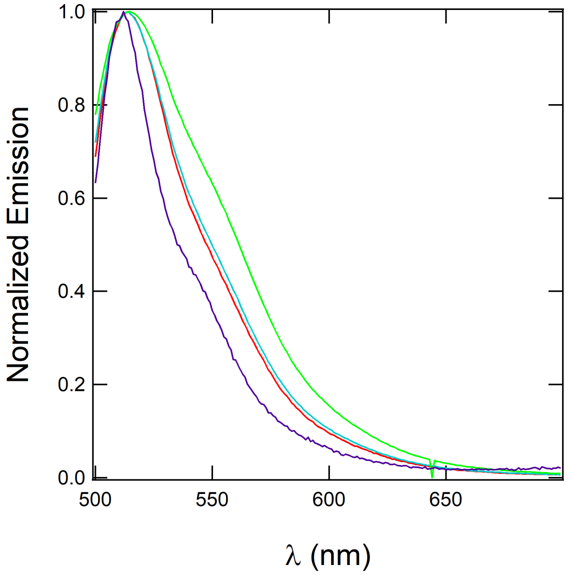

Figure S20: Normalized emission (λ_{exc} = 488 nm) spectra of 5 μM solutions in pH 7.4 PBS (50 mM sodium phosphate, 150 mM NaCl) of the 4 RSFPs : Dronpa (red), Dronpa-3 (blue), rsFastLime (green), and Dronpa-2 (violet); T = 298 K.

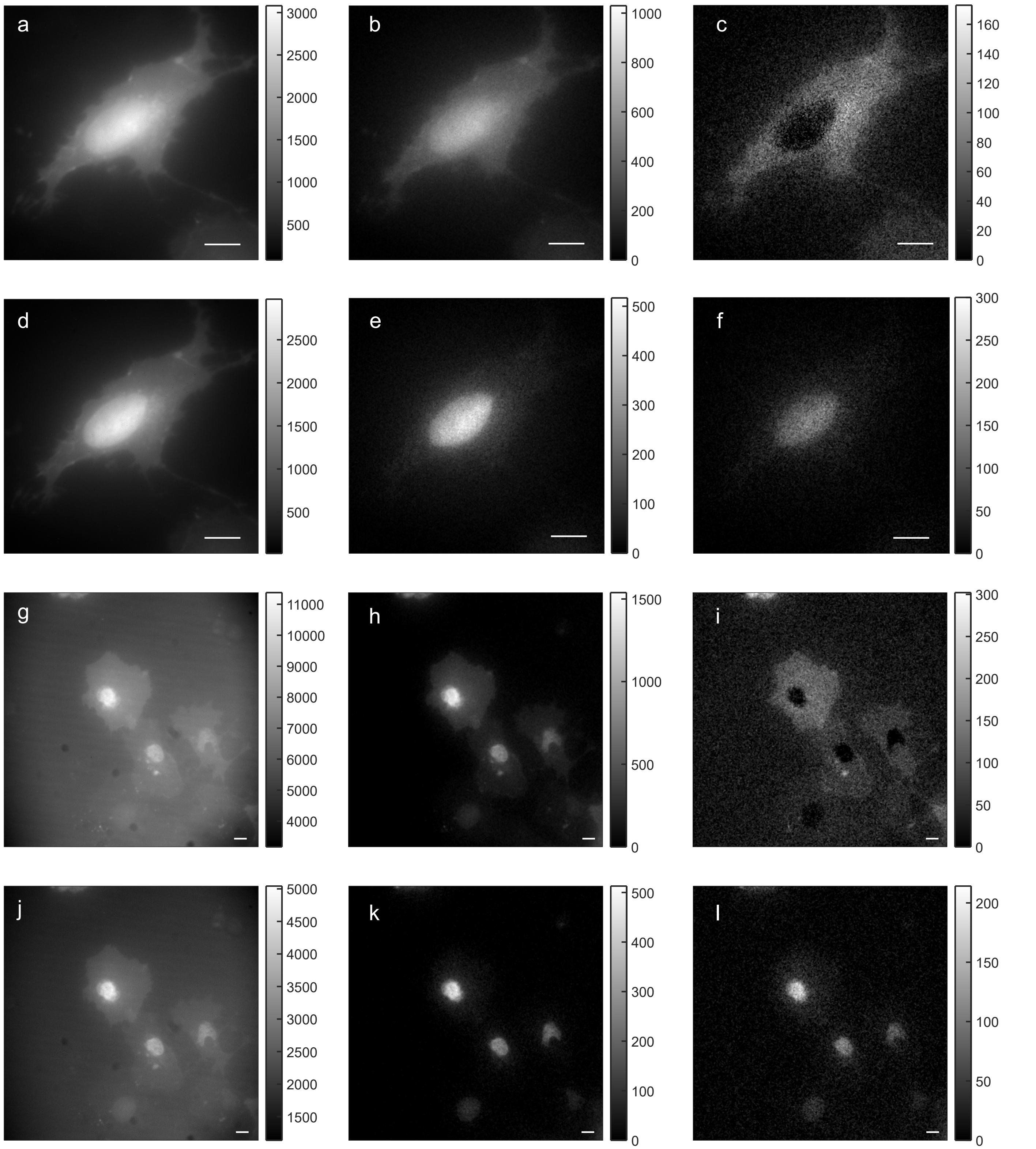

Figure S21: *HIGHLIGHT-2 discriminates distinct spectrally similar RSFPs in living cells.* Pre-HIGHLIGHT (**a**,**d**,**g**,**j**), HIGHLIGHT-1 (**b**,**e**,**h**,**k**), and HIGHLIGHT-2 (**c**,**f**,**i**,**l**) images of Dronpa-2 and rsFastLime. System: Fixed (**a**–**f**) and living (**g**–**l**) U2OS cells expressing Lyn11-rsFastLime (at the cell membrane) and H2B-Dronpa-2 (at the nucleus) . The images were recorded at 298 and 310 K for the fixed and living cells respectively. A median filter 3 $\times$ 3 was applied to obtain the final HIGHLIGHT-1, HIGHLIGHT-2 and HIGHLIGHT-3 images. The level of the HIGHLIGHT-n signals is displayed in the gray scale on the right of each figure. Scale bars: 10 μm. The control parameters are set at the resonance of HIGHLIGHT-1 for rsFASTLime (**a**–**c**, **g**–**i**) or Dronpa-2 (**d**–**f**, **j**–**l**). See Tab. S6 in Supporting Information for the acquisition conditions.

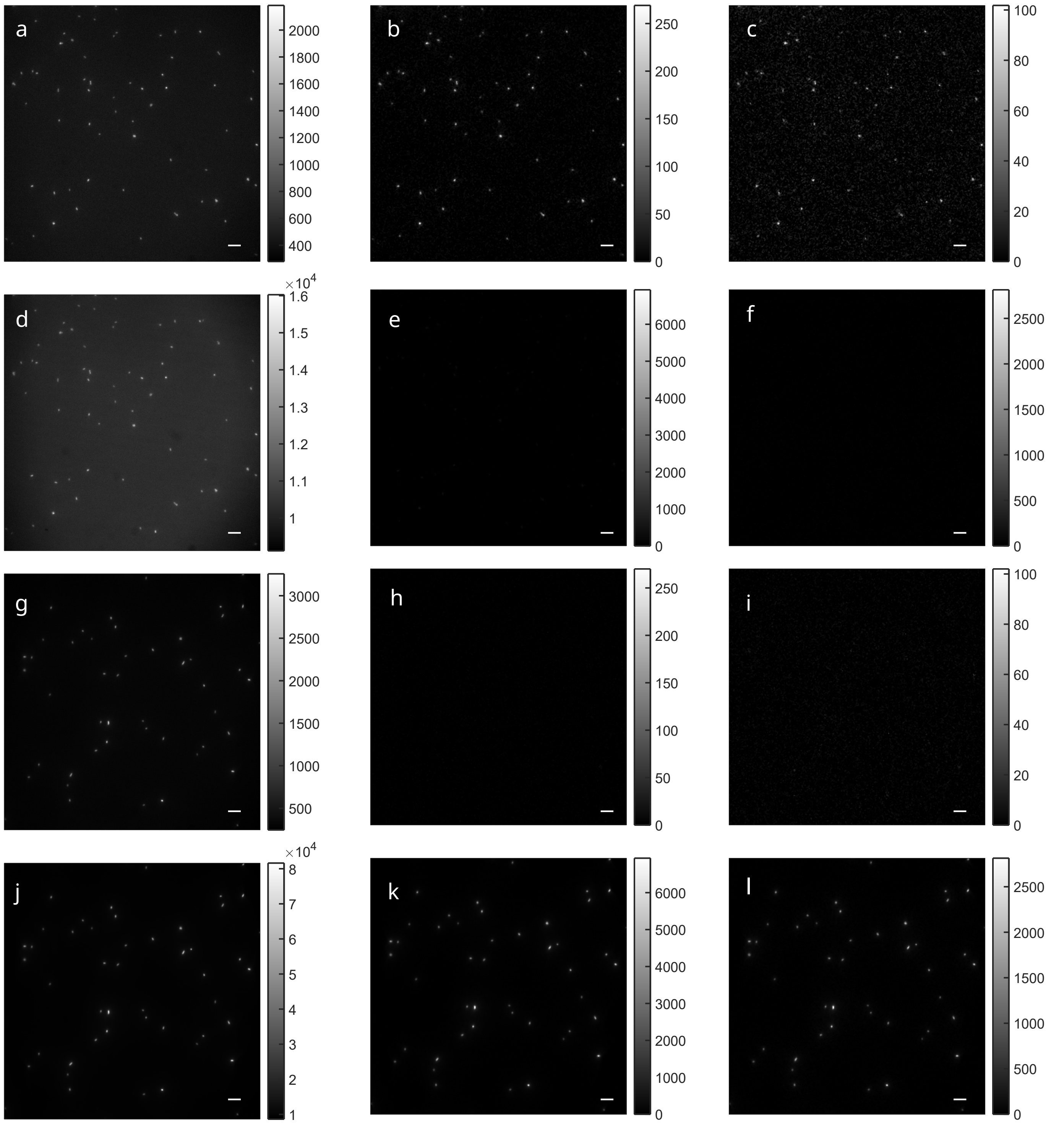

Figure S22: *HIGHLIGHT-2 discriminates distinct spectrally similar RSFPs in living bacteria*. Pre-HIGHLIGHT (**a**,**d**,**g**,**j**), HIGHLIGHT-1 (**b**,**e**,**h**,**k**), and HIGHLIGHT-2 (**c**,**f**,**i**,**l**) images of Dronpa-2 and Dronpa. System: Living E. Coli cells expressing Dronpa-2 (**a**–**f**) or Dronpa (**g**–**l**) . The images were recorded at 298 K.A median filter 3 $\times$ 3 was applied to obtain the final HIGHLIGHT-1, HIGHLIGHT-2 and HIGHLIGHT-3 images. The level of the HIGHLIGHT-n signals is displayed in the gray scale on the right of each figure. Scale bars: 10 μm. The control parameters are set at the resonance of HIGHLIGHT-1 Dronpa-2 (**a**–**c**, **g**–**i**) or Dronpa (**d**–**f**, **j**–**l**). See Tab. S6 in Supporting Information for the acquisition conditions.

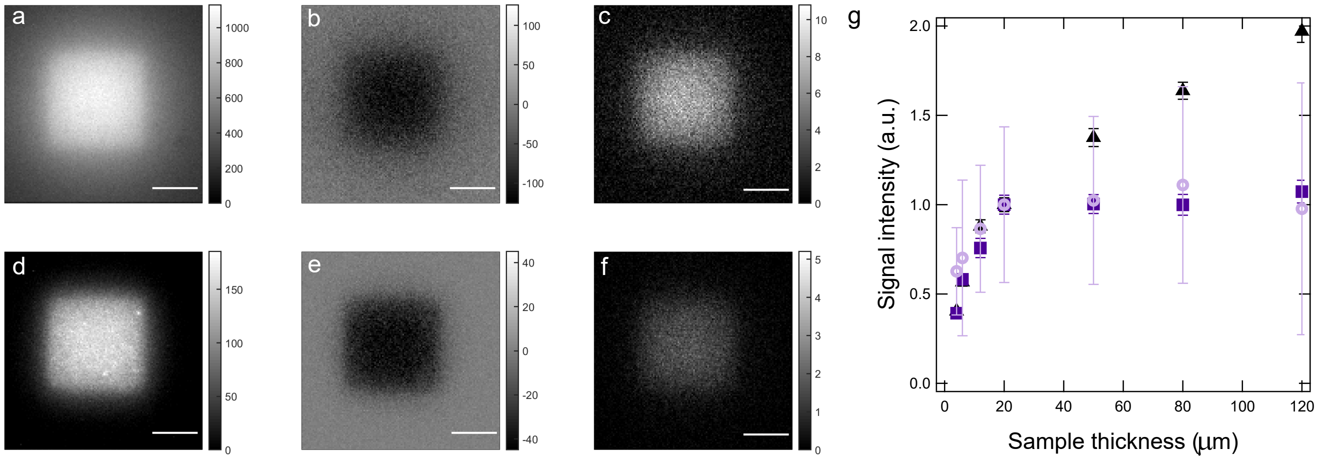

Figure S23: *HIGHLIGHT capability to enhance spatial contrast in non-homogeneous light profiles*. Pre-HIGHLIGHT (**a**,**d**), HIGHLIGHT-1 (**b**,**e**), -2 (**c**,**f**) images of a 10-μM Dronpa-2 solution of thickness 120 μm (**a**–**c**) and 4 μm (**d**–**f**) obtained with an optical setup using coaxial light profiles spatially decaying over 10 μm from a homogeneously illuminated 15-μm edge octahedron. Scale bar: 10 μm; **g**: Pre-HIGHLIGHT (black triangles), HIGHLIGHT-1 (deep violet squares), -2 (light violet circles) signals versus thickness z of the sample for a 10-μM Dronpa-2 solution. The mean signal intensities and the error bars are obtained using spatial averages of the images over a 6-μm square. See Tab. S6 in Supporting Information for the acquisition conditions with sine-wave light at $\lambda_2 = 405$ nm and constant light at $\lambda_1 = 480$ nm.

References

[1] S. Kredel, K. Nienhaus, F. Oswald, M. Wolff, S. Ivanchenko, F. Cymer, A. Jeromin, F. J. Michel, K.-D. Spindler, R. Heilker, G. U. Nienhaus, J. Wiedenmann, *Chem. Biol.* **2008**, *15*, 224–233.

[2] J. Querard, A. Gautier, T. Le Saux, L. Jullien, *Chem. Sci.* **2015**, *6*, 2968–2978.

[3] J. Querard, R. Zhang, Z. Kelemen, M.-A. Plamont, X. Xie, R. Chouket, I. Roemgens, Y. Korepina, S. Albright, E. Ipendey, M. Volovitch, H. L. Sladitschek, P. Neveu, L. Gissot, A. Gautier, J.-D. Faure, V. Croquette, T. Le Saux, L. Jullien, *Nat. Commun.* **2017**, *8*, 969.

Author Contribution

Conceptualization, A.Le., L.J. and T.L.S.; Methodology, A.P.-T., A.Le., V. C., L.J. and T.L.S.; Softwares, A.P.-T., A.La., A.Le., V.C. and T.L.S.; Formal analysis, A.P.-T., R.C., R. Z., A.Le., L.J. and T.L.S.; Investigation, A.P.-T., R.C., R. Z., A. E., and T.L.S.; Resources, A.P.-T., R.C., R. Z., A.Le., and T.L.S.; Writing – Original Draft, A.P.-T., R.C., R. Z., A.La., A.Le., L. J., and T.L.S.; Writing-Review & Editing, A.P.-T., R.C., A. E., A.Le., V. C., L. J., and T.L.S.; Funding Acquisition, A.E., A.Le., L.J. and T.L.S.
